# Genetic diversity and population structure of six autochthonous pig breeds from Croatia, Serbia, and Slovenia

**DOI:** 10.1186/s12711-022-00718-6

**Published:** 2022-04-28

**Authors:** Minja Zorc, Dubravko Škorput, Kristina Gvozdanović, Polona Margeta, Danijel Karolyi, Zoran Luković, Krešimir Salajpal, Radomir Savić, Maria Muñoz, Samuele Bovo, Ivona Djurkin Kušec, Čedomir Radović, Goran Kušec, Marjeta Čandek Potokar, Peter Dovč

**Affiliations:** 1 Department of Animal Science, University of Ljubljana, Biotechnical Faculty, Jamnikarjeva 101, 1000 Ljubljana, Slovenia; 2 University of Zagreb, Faculty of Agriculture, Svetošimunska cesta 25, 10000 Zagreb, Croatia; 3 Josip Juraj Strossmayer University of Osijek, Faculty of Agrobiotechnical Sciences Osijek, Vladimira Preloga 1, 31000 Osijek, Croatia; 4 Institute of Animal Sciences, University of Belgrade, Faculty of Agriculture, Nemanjina 6, 11080 Zemun, Belgrade, Serbia; 5 Departamento Mejora Genética Animal, INIA, Crta. de La Coruña, km. 7,5, 28040 Madrid, Spain; 6 Department of Agricultural and Food Sciences, Division of Animal Sciences, University of Bologna, 40126 Bologna, Italy; 7 Institute for Animal Husbandry, Autoput 16, 11080 Zemun, Belgrade, Serbia; 8 Agricultural Institute of Slovenia, Hacquetova ulica 17, 1000 Ljubljana, Slovenia

## Abstract

**Background:**

The importance of local breeds as genetic reservoirs of valuable genetic variation is well established. Pig breeding in Central and South-Eastern Europe has a long tradition that led to the formation of several local pig breeds. In the present study, genetic diversity parameters were analysed in six autochthonous pig breeds from Slovenia, Croatia and Serbia (Banija spotted, Black Slavonian, Turopolje pig, Swallow-bellied Mangalitsa, Moravka and Krskopolje pig). Animals from each of these breeds were genotyped using microsatellites and single nucleotide polymorphisms (SNPs). The results obtained with these two marker systems and those based on pedigree data were compared. In addition, we estimated inbreeding levels based on the distribution of runs of homozygosity (ROH) and identified genomic regions under selection pressure using ROH islands and the integrated haplotype score (iHS).

**Results:**

The lowest heterozygosity values calculated from microsatellite and SNP data were observed in the Turopolje pig. The observed heterozygosity was higher than the expected heterozygosity in the Black Slavonian, Moravka and Turopolje pig. Both types of markers allowed us to distinguish clusters of individuals belonging to each breed. The analysis of admixture between breeds revealed potential gene flow between the Mangalitsa and Moravka, and between the Mangalitsa and Black Slavonian, but no introgression events were detected in the Banija spotted and Turopolje pig. The distribution of ROH across the genome was not uniform. Analysis of the ROH islands identified genomic regions with an extremely high frequency of shared ROH within the Swallow-bellied Mangalitsa, which harboured genes associated with cholesterol biosynthesis, fatty acid metabolism and daily weight gain. The iHS approach to detect signatures of selection revealed candidate regions containing genes with potential roles in reproduction traits and disease resistance.

**Conclusions:**

Based on the estimation of population parameters obtained from three data sets, we showed the existence of relationships among the six pig breeds analysed here. Analysis of the distribution of ROH allowed us to estimate the level of inbreeding and the extent of homozygous regions in these breeds. The iHS analysis revealed genomic regions potentially associated with phenotypic traits and allowed the detection of genomic regions under selection pressure.

**Supplementary Information:**

The online version contains supplementary material available at 10.1186/s12711-022-00718-6.

## Background

In the past, the development of livestock production was mainly based on the formation of local breeds that were well adapted to specific conditions and rearing practices. However, in the second half of the twentieth century, the lower economic performance of these breeds when raised under intensive production conditions resulted in a significant reduction of local pig breed populations, which were replaced by modern, highly productive pig breeds adapted to farm conditions and constraints [1]. In recent years, there has been growing awareness of the importance of local breeds in terms of adaptive traits, as a reservoir of valuable genetic variation with potential for more sustainable added-value oriented pork production, and because of their historical and cultural value [2–4]. Local breeds are considered essential for maintaining future breeding options since genetic diversity is important for improving traits of interest [1]. Pig breeding in Central and South-Eastern Europe has a long and rich tradition, which led to the formation of several local pig breeds. From a historical point of view, pig breeds from Croatia, Serbia, and Slovenia shared a common breeding area that was part of the Austro-Hungarian Empire and later Yugoslavia. Today, these local pig breeds are subject to conservation programmes in these countries.

In the present study, six local pig breeds: Banija spotted, Black Slavonian and Turopolje pig from Croatia, Mangalitsa (Swallow-bellied Mangalitsa) and Moravka from Serbia, and Krskopolje pig from Slovenia, were analysed. Most of these breeds have low to moderate production performances [4]. While the origin of the Black Slavonian and Banija spotted breeds has been reconstructed [5, 6], no reliable data on the history of the four other breeds are available. The influence of modern pig breeds (Landrace and Yorkshire) is observed in the Krskopolje pig [7] and Banija spotted breeds [6], while the contribution of some older breeds (Berkshire) is detected in the Black Slavonian [8] and Moravka breeds [9]. The Turopolje pig breed is one of the oldest European pig breeds that originated in the Middle Ages [10]. The main characteristics of the six pig breeds analysed here are a high fat accumulation, including intramuscular fat, and a high suitability for processing traditional meat products [4, 11]. Most of these breeds are mixed-type breeds with only Mangalitsa and Turopolje pig being fat-type breeds [10, 11].

The genetic diversity and the relationships between the six breeds analysed in this study were previously estimated using microsatellite, mitochondrial DNA (mtDNA) and single nucleotide polymorphism (SNP) markers. Analyses based on microsatellite markers [12, 13] and pedigree data [14, 15] indicated that the Black Slavonian, Swallow-bellied Mangalitsa, Turopolje and Banija spotted pig breeds are generally well-differentiated. Analysis of the Hungarian population of Mangalitsa pigs that were genotyped at 10 microsatellite loci identified three clusters within this breed (Swallow-bellied, Red and Blond) [16] but analyses based on mtDNA markers, could not separate it into subpopulations [17]. Analysis of the Krskopolje pig using 11 microsatellite loci revealed three clusters that were clearly separated from the German Sattelschwein and Slovenian Landrace breeds [18]. Based on both microsatellite [13, 19, 20] and SNP [21, 22] analyses, the Turopolje pig was shown to have a low genetic diversity and a high level of inbreeding. The analysis of 39 SNPs within 33 genes associated with quantitative traits revealed a relatively high level of observed and expected heterozygosity in the Moravka and Krskopolje pig breeds [23]. Genotyping analysis based on a 60 k SNP array showed that the Black Slavonian breed was positioned close to the UK/North American cluster of breeds, and the Turopolje pig clustered with the Mediterranean breeds [21]. Bovo et al. [24] reported the construction of a neighbour-joining tree based on ${\mathrm{F}}_{\mathrm{ST}}$ values from pool-Seq data for various European pig breeds that showed that the Krskopolje and Swabian-Hall pig breeds were on the same branch and that the Swallow-bellied Mangalitsa and Black Slavonian breeds clustered together with the wild boar.

The six autochthonous pig breeds analysed here have been subjected to different selective pressures in the past and are currently managed under conservation programmes. The Banija spotted breed is a newly recognised autochthonous breed and was genotyped using a high-density SNP panel within the frame of our study. Since the main goal of conservation programmes is to maintain genetic diversity, we used pedigree, microsatellite and SNP data to comprehensively investigate the level and patterns of genetic diversity and to infer population structure and isolation by distance for these six breeds. We also compared the performance of two systems of genetic markers (microsatellites and SNPs) and of pedigree data applied to the animal material available here.

## Methods

### Sample collection

**Fig. 1:**
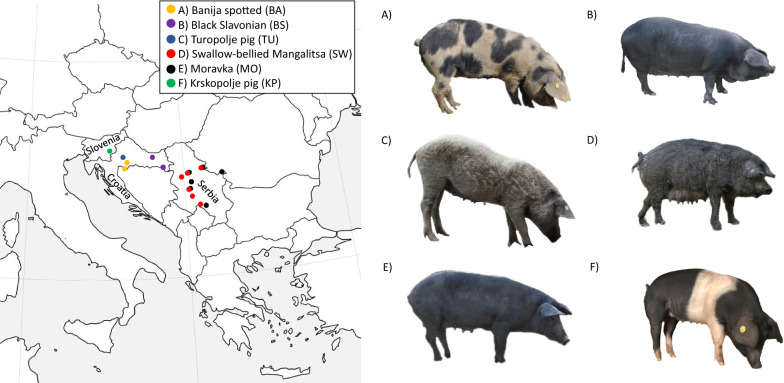
Map of sampling locations of the six autochthonous pig breeds from Croatia, Serbia and Slovenia

The six autochthonous pig breeds included in our study (see Fig. 1) were: three breeds from Croatia, Banija spotted (Banijska šara), Black Slavonian (Crna slavonska svinja), and Turopolje pig (Turopoljska svinja), two breeds from Serbia, Swallow-bellied Mangalitsa (Lasasta mangulica) and Moravka (Moravka), and one Slovenian breed, Krskopolje pig (Krškopoljski prašič).

Banija spotted (Fig. 1a) is the latest recognised pig breed in Croatia and is currently in the process of breed valorisation. It is characterised by a large body frame, a white coat with black spots and a good fertility rate [6]. Black Slavonian (Fig. 1b) is a medium-sized breed characterised by a black coat colour and a high intramuscular fat (IMF) content, which is important for the quality of pork products [5]. Moravka (Fig. 1e) is a medium-sized pig breed with black pigmented skin and straight, smooth dark hair and is used for meat and fat production [9]. Krskopolje pig (Fig. 1f) is a middle to large-sized breed with a black coat and white belt over the shoulders. Its IMF content is lower (2.0–4.3%) than for the other analysed breeds [25]. Fatty type breeds are represented by the medium-sized Turopolje pig (Fig. 1c) and the Swallow-bellied Mangalitsa (Fig. 1d) breeds. Turopolje pig is one of the oldest breeds in Europe, with white or grey curly hair and sporadic black spots and is known for its modest production but high resilience and outdoor foraging capability [10]. Swallow-bellied Mangalitsa is a medium-sized breed, known for its woolly coat, with a relatively small body frame, low fertility and good adaptability [11].

### Workflow summary of the study

**Fig. 2:**
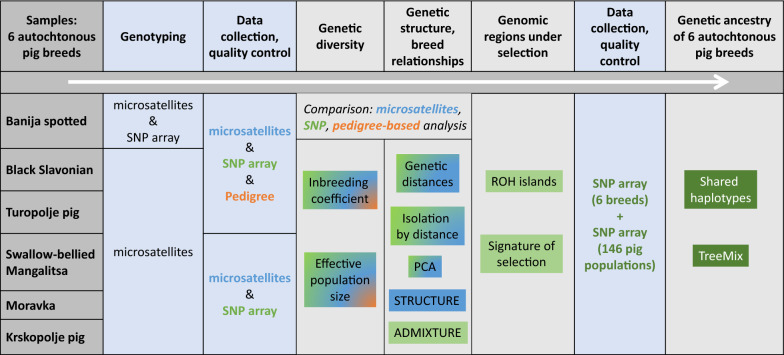
Workflow summary of the study. The diagram explains the workflow and the research methods applied to various types of data in this study. Analyses based on (1) microsatellite markers are marked in blue, (2) SNPs in green, and (3) pedigree data in orange. SNP array analyses that include only the six autochthonous pig breeds, are marked with light green, and those that were performed on a merged dataset with 146 additional pig populations are marked with dark green

A workflow summary of the study is presented in Fig. 2. We used several combinations of different datasets: two types of markers, microsatellites and SNPs, to estimate the genetic diversity and genetic structure of the six pig breeds, and pedigree data that were available for three of the breeds, Banija spotted, Black Slavonian and Turopolje, which allowed us to compare estimates of population parameters from three types of data for these three breeds. The genome-wide scans for putative traces of past selection events were performed using SNP array data [22]. In addition, a previously reported SNP array dataset [26], including 146 pig populations (autochthonous breeds, commercial breeds, and wild boars), was added to our genotyping dataset to analyse the relationships between these 146 pig populations and our six breeds.

### Pedigree analysis

Pedigree records for the Banija spotted, Black Slavonian and Turopolje pig populations were provided by the Croatian Ministry of Agriculture (Table 1). The CFC software package [27] was used to detect the basic pedigree structure, and the individual inbreeding coefficient ($\mathrm{F}$), which is defined as the probability of identity-by-descent [28], was calculated from the pedigree data. The effective population size (${\mathrm{N}}_{\mathrm{e}}$), defined as the number of individuals that would generate the current level of inbreeding, was calculated as in [29]:

**Table 1:** Pedigree structure for the Banija spotted, Black Slavonian and Turopolje pig breeds

Breed	Number of individuals	Mean equivalent generation	Mean complete generation
Banija spotted	221	2.00	1.50
Black Slavonian	5732	2.12	1.30
Turopolje pig	947	1.64	1.15

$${\mathrm{N}}_{\mathrm{e}}=\frac{1}{2\mathrm{ \Delta F}}.$$

The inbreeding rate ($\mathrm{\Delta F}$) was computed for each generation [29] as:

$$\Delta \mathrm{F}=\frac{{\mathrm{F}}_{\mathrm{t}} - {\mathrm{F}}_{\mathrm{t}-1}}{1-{\mathrm{F}}_{\mathrm{t}-1}},$$

where ${\mathrm{F}}_{\mathrm{t}}$ and ${\mathrm{F}}_{\mathrm{t}-1}$ are the average inbreeding coefficients for the current and the previous generation, respectively, as implemented in the ENDOG software [30].

Quality and integrity of the pedigree information were assessed by: (1) the number of complete generations, defined as the number of generations that separate the offspring from the most distant known ancestor for which its ${2}^{\mathrm{g}}$ ancestors are known ($\mathrm{g}$ is the number of generations); and (2) the number of complete equivalent generations, defined as the sum over all known ancestors of the terms, calculated as the sum of ${(1/2)}^{\mathrm{n}}$, where $\mathrm{n}$ is the number of generations that separate the individual from each known ancestor.

### Microsatellite genotyping

In total, 214 blood samples were obtained for the six breeds: Banija spotted (n = 24), Black Slavonian (n = 47), Turopolje pig (n = 17), Swallow-bellied Mangalitsa (n = 45), Moravka (n = 47) and Krskopolje pig (n = 34). Genomic DNA was extracted from the blood samples using the GeneJET™ Genomic DNA Purification Kit (Thermo Scientifc™) according to the manufacturer’s protocol. All animals were genotyped at 25 microsatellite loci (S0026, S0155, S0005, Sw24, Sw632, Swr1941, Sw2410, Sw830, S0355, Sw9366, S0218, S0228, Sw240, Sw2406, Sw122, Sw857, S0097, Sw72, S0226, S0090, Sw911, S0002, Sw2008, Sw1067, and S0101) selected from the ISAG/FAO list [31], and grouped into three multiplex PCR reactions (see Additional file 1: Table S1). Multiplex PCR amplification was performed according to Margeta et al. [32], and Gvozdanović et al. [33]. PCR products were analysed using the GeneScan350 ROX internal standard size marker on an ABI Prism™ 310 Genetic Analyzer (PE Applied Biosystems). Since the Sw2008 microsatellite was not informative for the Turopolje and Banija spotted pig breeds, it was excluded from further analysis.

### Microsatellite data analysis

Genetic variability was measured across the remaining 24 microsatellites for each population using the F statistic parameters (${\mathrm{F}}_{\mathrm{IS}}$, ${\mathrm{F}}_{\mathrm{ST}}$, and ${\mathrm{F}}_{\mathrm{IST}}$), number of alleles (${\mathrm{N}}_{\mathrm{A}}$), number of effective alleles (${\mathrm{N}}_{\mathrm{A}/\mathrm{E}}$), observed (${\mathrm{H}}_{\mathrm{obs}}$) and expected (${\mathrm{H}}_{\mathrm{exp}}$) heterozygosity using the GENETIX 4.03 software [34]. Estimates of effective population size (${\mathrm{N}}_{\mathrm{e}}$) and polymorphism information content (PIC) per locus were calculated using the NeEstimator v.2.0 software [35]. The average number of null alleles, private alleles and percentage of polymorphic loci were calculated using the GenAlEx 6.5 software [36]. Genetic diversity statistics across loci were calculated using the *poppr* [37] library for R [38]. Principal component analysis (PCA) was performed using the *ade4* [39] package (version 1.7.15) in R [38]. 3D PCA was visualised using the *scatter3* function in the MATLAB R2020b software http://www.mathworks.com. We evaluated the population structure by using the STRUCTURE v.2.3.4 software [40] and setting run-length to 50,000 iterations followed by 100,000 Markov chain Monte Carlo (MCMC) iterations under an admixture model with correlated allele frequencies. The program was run within 10 simulations for each K value from 1 to 9. The number of genetic clusters (K) that were best supported by the data was evaluated according to Evanno et al. [41]. The STRUCTURE results were visualised using the CLUMPAK software [42].

### SNP array genotyping of the Banija spotted breed

The quality of the genomic DNA extracted from the 24 Banija spotted individuals (see above) was assessed using a NP80 NanoPhotometer (Implen GmbH, Munich, Germany). SNPs were genotyped using the GeneSeek Genomic Profiler (GGP) Porcine 80K Bead Chip (Neogen, Lincoln, NE, USA).

### Construction and quality control of SNP datasets

Two SNP datasets obtained from three different SNP arrays (see Table 2) were used in the current study: the first dataset (six local pig breeds) contained (A) 24 Banija spotted pig samples genotyped for this study (see above section) and (B) 275 samples from the Black Slavonian, Turopolje pig, Swallow bellied Mangalitsa, Moravka and Krskopolje pig breeds. The second dataset (6 local + 146 other pig breeds) contained six local pig breeds (A and B) and (C) 2113 samples belonging to 146 pig populations downloaded from DRYAD (dryad.30tk6).

**Table 2:** SNP array data used in this study

Breeds in each SNP dataset	SNP array	References
A. Banija spotted (n = 24)	GeneSeek Genomic Profiler (GGP) Porcine Bead Chip 80 K array (Neogen, Lincoln, NE, USA)	This study
B. Black Slavonian, Turopolje pig, Swallow-bellied Mangalitsa, Moravka, Krskopolje pig	GeneSeek SNP70 BeadChip (Neogen, Lincoln, NE, USA)	[22]
C. Domestic, wild, feral and outgroup suids (146 pig populations, 2113 samples)	Illumina Porcine SNP60 BeadChip (Illumina Inc., San Diego, CA, USA)	[26]

Data merging and quality control procedures were performed using the SNP & Variation Suite v8.8.3 (Golden Helix, Inc., Bozeman, MT, www.goldenhelix.com). SNPs located on the sex chromosomes and SNPs with a call rate lower than 90% were removed, and the samples with a call rate lower than 95% and the duplicated samples (detected using the SVS function “identity by descent estimation”) were excluded. Finally, in the first dataset, 258 samples and 57,781 SNPs were retained: Banija spotted (n = 24), Black Slavonian (n = 41), Turopolje pig (n = 47), Swallow-bellied Mangalitsa (n = 50), Moravka (n = 50) and Krskopolje pig (n = 46), and in the second dataset 2350 samples and 37,371 SNPs (from two different SNP panels that share 40,753 SNPs) remained for further analyses. The genomic coordinates of SNPs were based on the pig genome assembly Sscrofa 11.1.

### Genetic diversity parameters and effective population size inferred from SNP data

Population genetic statistics were calculated using the module Stacks Populations [43]. Nei’s genetic distances between breeds and breed-pairwise genetic differentiation based on Cockerham and Weir ${\mathrm{F}}_{\mathrm{ST}}$ were calculated and visualised using the JMP® software, Version 9, SAS Institute Inc., Cary, NC, 1989–2019. Effective population size (${\mathrm{N}}_{\mathrm{e}}$) for each breed was estimated by SNP-based linkage disequilibrium (LD) analysis with the SNeP software [44]. MAF filtering was set to 0.05, the sample size was corrected, and the minimum and maximum distances between the analysed SNPs were set to 0.05 and 4.00 Mb, respectively. The Sved and Feldman approximation was used to correct the recombination rate [45].

### Comparison between two marker systems: SNPs and microsatellites

To compare the two marker systems (microsatellites and SNPs) used for the six breeds analysed in this study, the informativeness for assignment (${\mathrm{I}}_{\mathrm{n}}$) [46] implemented in the diveRsity [47] package for R [38] was calculated. The correlation between pairwise estimates of ${\mathrm{F}}_{\mathrm{ST}}$ obtained from microsatellites and SNPs was tested by a Mantel test implemented in the *ecodist* [48] library for R. The Ggplot2 tool [49] was used to visualise the distribution of the informativeness of the two marker types and their pairwise ${\mathrm{F}}_{\mathrm{ST}}$ correlations.

### Analysis of runs of homozygosity

Runs of homozygosity (ROH) were identified separately for each population using the SNP and Variation Suite v8.9.0. The minimum run length was set to 1.0 Mb, the minimum number of homozygous SNPs within a run was set to 25, and a maximum of five SNPs with missing genotype and no heterozygous SNPs were allowed within a run. ROH were grouped by length: short (0.5 to 2.5 Mb), medium (2.5 to 5.0 Mb) and long (longer than 5 Mb). Genomic inbreeding coefficients (${\mathrm{F}}_{\mathrm{ROH}}$) were defined as the proportion of the autosomal genome covered by ROH [50]. The autosomal genome length was set to 2,330,828,850 bp. ROH islands defined as regions of the genome where individuals of the same breed share ROH were identified. A Manhattan plot of the occurrence of SNPs in ROH across individuals was visualised using the SNP and Variation Suite v8.9.0. Genes harbouring ROH islands were analysed using Ensembl, release 102 (http://nov2020.archive.ensembl.org/) and plotted on chromosome idiograms using the RIdeogram [51] library for R [38].

### Analysis of population structure

Principal component analyses (PCA) were performed using the *glPCA* function of the Adegenet package [52], version 2.1.3 for R [38]. We visualised the PCA using the *scatter3* function in MATLAB R2020. The SNP & Variation Suite v8.9.0 was used for pruning SNPs based on LD with default parameters (window size 50, increment 5, r^2^ threshold of 0.5), leaving 32,987 SNPs. To avoid bias related to sample size, we resized the dataset to a maximum of 24 randomly selected individuals per breed. Population structure was evaluated using the ADMIXTURE software [53]. The program was run for K values from 2 to 14. The number of genetic clusters that best matched the data was estimated through 20-fold cross-validation of the accuracy to correctly assign samples to clusters. The ADMIXTURE results were visualised using the CLUMPAK software [41].

### Isolation-by-distance

Isolation-by-distance between populations was assessed using the Mantel test [54], which was originally formulated as:

$${\mathrm{Z}}_{\mathrm{m}}=\sum_{\mathrm{i}-1}^{\mathrm{n}}\sum_{\mathrm{j}-1}^{\mathrm{n}}{\mathrm{g}}_{\mathrm{ij}}*{\mathrm{d}}_{\mathrm{ij}},$$

where ${\mathrm{g}}_{\mathrm{ij}}$ and ${\mathrm{d}}_{\mathrm{ij}}$ are the genetic and geographical distances between populations $\mathrm{i}$ and $\mathrm{j}$, respectively, considering $\mathrm{n}$ populations. To assess the correspondence between pairwise genetic (Nei) and geographical distances between breeds, the Mantel test implemented in *ecodist* package [47] in R [38] was used.

### Haplotype sharing analysis

Phasing and identity-by-descent (IBD) haplotype analysis for the dataset, comprising 152 pig populations (6 local + 146 other pig breeds), was performed using BEAGLE v4.1 [55]. To correct for local variations in recombination rate, the sex-averaged map of recombination rate in 1-Mb windows [56] of the pig genome was used. The minimum length of IBD shared haplotypes was set to 100 kb, three markers were trimmed from the end of each shared haplotype when testing for IBD, and the minimum LOD score for reported IBD was set to 2.5. The inferred IBD shared haplotypes were grouped into size categories: short i.e. less than 3 Mb, medium i.e. 3 to 7 Mb and long i.e. more than 7 Mb. For each of the analysed breeds, the average number of shared haplotypes with individuals from the other breeds was calculated for each haplotype size category.

### TreeMix analysis

The maximum likelihood algorithm implemented in TreeMix 1.13 was applied to detect traces of genetic admixture between the analysed pig breeds. The dataset for TreeMix analysis was constructed by including samples from the pig breeds for which shared haplotypes with any of the six local breeds from this study were detected. The TreeMix analysis was performed for 1 to 15 migration events (five iterations per migration edge). LD between SNPs was considered by grouping SNPs in blocks of 1000. The tree was rooted with wild boar samples from Finland. The best predictor for number of migration events was selected using the ‘optM function’ in the R package optM [57]. Then, a consensus tree including bootstrap node support was obtained by running TreeMix 100 times (m = 11) and using the BITE package for R [58].

### Signatures of selection

Analysis of signatures of selection was performed using phased SNP data. The *rehh* package [59] in R [38] was used to detect signatures of selection within populations using the integrated haplotype score (iHS). The putative signatures of selection detected by iHS were annotated using Ensembl, release 102, and visualised as a Manhattan plot using the *manhattanplot* function provided by *rehh* package. Genes within candidate signatures of selection were plotted on chromosome idiograms using the RIdeogram [51] library for R [38]. The chromosomes were coloured according to the estimates of genome-wide recombination rate in pig [56].

## Results

### Analysis of the microsatellite and SNP data

The microsatellite data set contained genotypes at 24 microsatellites for at least 17 animals of each of the six breeds analysed (n = 214 pigs). Population statistic parameters from the microsatellite panel were estimated for each population (Table 3). Significant deviation from Hardy–Weinberg equilibrium (p < 0.05) was detected only for the Turopolje pig, and a frequency of null alleles of 5.1% or less was detected for the six breeds. All the microsatellites included in the panel were highly polymorphic with a polymorphism information content (PIC) value ranging from 0.317 in the Turopolje pig to 0.631 in the Krskopolje pig. The average frequency of private alleles was highest in the Turopolje pig (0.118) and lowest in the Swallow-bellied Mangalitsa breed (0.011).

**Table 3:** Allele frequencies and informativeness of the microsatellite data for the six Balkan autochthonous pig breeds analysed

Breed	N	PIC	Null alleles	Pa	Polymorphic loci (%)
Banija spotted	24	0.601	0.051	0.046	100.00
Black Slavonian	47	0.607	0.001	0.046	100.00
Turopolje pig	17	0.317	0.001	0.118	95.83
Swallow-bellied Mangalitsa	45	0.539	0.035	0.011	100.00
Moravka	47	0.622	-0.004	0.030	100.00
Krskopolje pig	34	0.631	0.040	0.060	100.00

N—number of animals; PIC—polymorphism information content; Pa—average frequency of private alleles in the studied populations

After quality control of the SNP chip data, 258 samples with 57,781 SNPs remained for further analyses (Table 4). The SNP density on the array was equal to one SNP per 44.813 kb with a minimum and maximum gap of 13 and 1,059,766 bp, respectively. The overall nucleotide diversity ($\uppi$) ranged from 0.173 to 0.371, the average major allele frequency (P) ranged from 0.721 to 0.877, and the number of private alleles was smallest (4) in the Turopolje pig and largest (62) in the Moravka breed.

**Table 4:** Frequency of polymorphic sites and nucleotide diversity inferred by SNP data analysis in the six Balkan autochthonous pig breeds

Breed	N	NP	Polymorphic loci (%)	P (SD)	${\varvec{\uppi}}$(SD)
Banija spotted	24	52	97.237	0.721 (0.140)	0.371 (0.143)
Black Slavonian	41	17	96.944	0.743 (0.148)	0.342 (0.156)
Turopolje pig	47	4	62.600	0.877 (0.151)	0.173 (0.188)
Swallow-bellied Mangalitsa	50	6	84.457	0.808 (0.162)	0.260 (0.190)
Moravka	50	62	98.811	0.733 (0.142)	0.355 (0.144)
Krskopolje pig	46	47	97.772	0.723 (0.141)	0.365 (0.144)

N—number of animals; NP—number of private alleles in the studied populations; P—average major allele frequency; $\uppi$—an estimate of nucleotide diversity

### Comparison between SNP and microsatellite markers

**Fig. 3:**
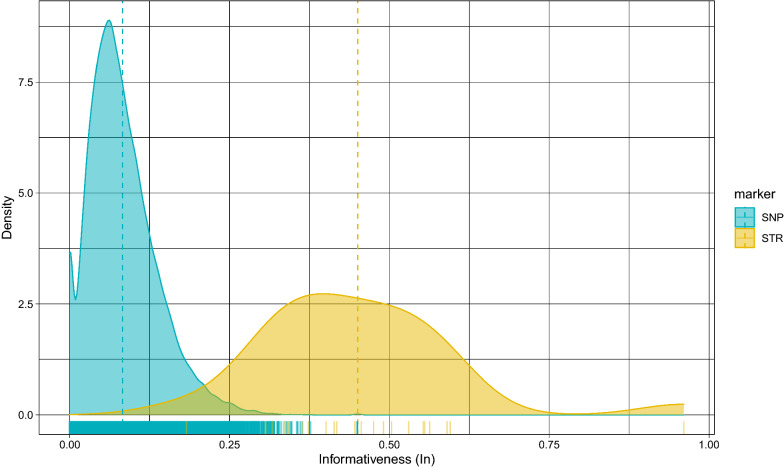
Distributions of informativeness for assignment (${\mathrm{I}}_{\mathrm{n}}$) of microsatellites and SNPs presented by the density plot. The mean values of informativeness for assignment (${\mathrm{I}}_{\mathrm{n}}$) are indicated with dashed lines

Informativeness of the microsatellite and SNP panels was evaluated for the studied populations (Fig. 3). For the microsatellite panel, the highest informativeness for assignment to each population (${\mathrm{I}}_{\mathrm{n}}$) was observed for microsatellite S0005 (${\mathrm{I}}_{\mathrm{n}}$ = 0.961) with 18 alleles, the mean ${\mathrm{I}}_{\mathrm{n}}$ was equal to 0.45, and on average, there were 9.79 alleles per microsatellite. Microsatellite S0005 also had the highest Simpson’s index (1-D = 0.91), Shannon–Wiener index (H = 2.55), Nei’s unbiased gene diversity (GD = 0.91), and polymorphism information content value (PIC = 0.899) (see Additional file 2: Table S2). For the SNP panel, the mean I_n_ was equal to 0.083 and the highest ${\mathrm{I}}_{\mathrm{n}}$ (${\mathrm{I}}_{\mathrm{n}}$ = 0.45) was found for 15 SNPs distributed on 10 chromosomes (see Additional file 3: Table S3). Four SNPs did not map to the reference genome (Sscrofa11.1).

**Fig. 4:**
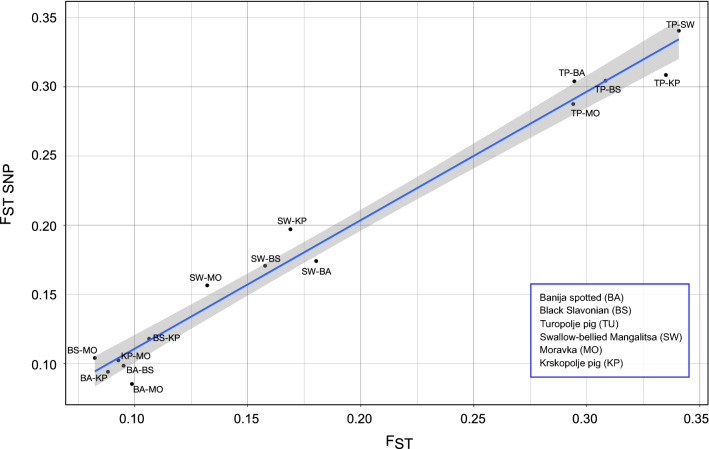
Scatter plot comparison of pairwise ${\mathrm{F}}_{\mathrm{ST}}$ estimated with 57,781 SNPs and with 24 microsatellites among the six autochthonous pig breeds. The graph shows the obtained data, the fitted linear regression line (blue) and its 95% confidence interval (grey zone). The labels on points show each pair of breeds used to estimate ${\mathrm{F}}_{\mathrm{ST}}$

The ${\mathrm{F}}_{\mathrm{ST}}$ values between breeds inferred from both marker types were calculated (Fig. 4) and (see Additional file 4: Table S4), and the Mantel test revealed a significant correlation (${\mathrm{r}}_{\mathrm{m}}$ < 0.05) between the estimates. Breed-pairwise ${\mathrm{F}}_{\mathrm{ST}}$ values ranged from 0.082 to 0.341 for microsatellites and from 0.079 to 0.351 for SNPs.

### Summary of population genetics statistics

Parameters of genetic variability were calculated for both panels (24 microsatellites and 57,781 SNPs) (Table 5). The differences between expected and observed heterozygosities for both types of markers were small, but the absolute values of heterozygosity differed considerably between microsatellite-based and SNP-based values. For both marker types, the level of heterozygosity was lowest for the Turopolje pig, followed by the Swallowed-bellied Mangalitsa breed.

**Table 5:** Population genetics parameters inferred from the microsatellite (STR) and SNP data for the six Balkan autochthonous pig breeds

Breed	H_exp STR_	H_obs STR_	F_ST STR_	F_IT STR_	N_A STR_	N_A/E_	H_exp SNP_	H_obs SNP_
Banija spotted	0.640	0.595	0.170	0.189	5.750	3.339	0.363	0.363
Black Slavonian	0.651	0.655	0.177	0.213	6.292	3.366	0.338	0.342
Turopolje pig	0.359	0.392^a^	0.123	0.155	3.125	1.814	0.171	0.188
Swallow-bellied Mangalitsa	0.594	0.562	0.157	0.170	4.583	2.840	0.258	0.256
Moravka	0.660	0.667	0.185	0.220	6.542	3.419	0.352	0.348
Krskopolje pig	0.679	0.657	0.171	0.196	5.750	3.511	0.361	0.366

H_exp_—expected heterozygosity; H_obs_—observed heterozygosity; F_ST_—deviation from Hardy–Weinberg equilibrium between the studied populations; F_IT_—deviation from Hardy–Weinberg equilibrium within each investigated population; N_A **STR**_—number of alleles for the microsatellites; N_A/E_—number of effective microsatellite alleles

^a^Significant at p < 0.01

### Inbreeding

**Fig. 5:**
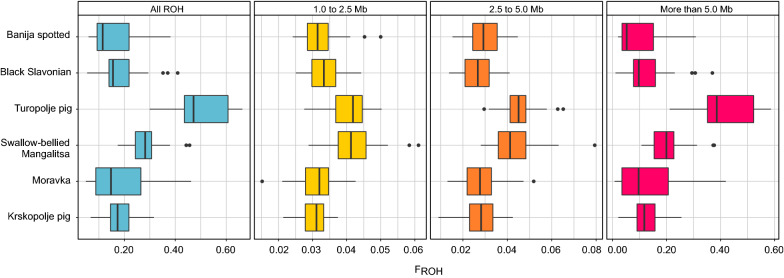
Proportion of the autosomal genome in ROH (${\mathrm{F}}_{\mathrm{ROH}}$) for each of the six autochthonous pig breeds analysed considering all, short (between 1.0 and 2.5 Mb), medium (between 2.5 and 5.0 Mb) and long (longer than 5 Mb) ROH

Genomic inbreeding was estimated using inbreeding coefficients calculated from pedigree ${\mathrm{F}}_{\mathrm{PED}}$, microsatellite ${\mathrm{F}}_{\mathrm{IS STR}}$, SNP data ${\mathrm{F}}_{\mathrm{IS SNP}}$, and ${\mathrm{F}}_{\mathrm{ROH}}$ (Table 6). ${\mathrm{F}}_{\mathrm{PED}}$ was highest in the Turopolje breed (0.038), followed by the Black Slavonian (0.020) and Banija spotted breeds (0.017), and the ranking of inbreeding estimates inferred from ROH (${\mathrm{F}}_{\mathrm{ROH}})$ was the same as that of ${\mathrm{F}}_{\mathrm{PED}}$ (Fig. 5).

**Table 6:** Inbreeding coefficients for the six Balkan autochthonous pig breeds

Breed	F_PED_	F_IS STR_	F_IS SNP_	F_ROH_
Banija spotted	0.017	0.024	0.020	0.160
Black Slavonian	0.020	0.044	0.001	0.183
Turopolje pig	0.038	-0.039	− 0.039	0.508
Swallow-bellied Mangalitsa	–	0.024	0.013	0.285
Moravka pig	–	0.044	0.021	0.179
Krskopolje pig	–	0.032	– 0.002	0.185

Inbreeding coefficients: ${\mathrm{F}}_{\mathrm{PED}}$ —based on pedigree, ${\mathrm{F}}_{\mathrm{IS STR}}$—based on microsatellites, ${\mathrm{F}}_{\mathrm{IS SNP}}$ —based on SNPs, ${\mathrm{F}}_{\mathrm{ROH}}$ —derived from ROH

The average length and number of identified ROH differed considerably among the six studied breeds (Table 7), but the majority of ROH were short (1.0 to 2.5 Mb).

**Table 7:** Summary statistics of ROH based on length classes for the six Balkan autochthonous pig breeds

	Banija spotted	Black Slavonian	Turopolje pig	Swallow-bellied Mangalitsa	Moravka	Krskopolje pig
Mean number of ROH (SD)						
All (> 1.0 Mb)	84.000 (19.600)	87.585 (14.036)	146.915 (12.092)	120.620 (12.857)	81.660 (15.387)	83.717 (9.609)
Short (1.0 to 2.5 Mb)	46.750 (9.294)	48.854 (7.663)	57.915 (7.709)	61.160 (9.980)	45.180 (7.176)	44.652 (5.372)
Medium (2.5 to 5.0 Mb)	19.875 (5.448)	17.854 (4.752)	29.532 (4.525)	28.340 (6.536)	19.020 (5.460)	18.109 (4.941)
Long (> 5.0 Mb)	17.375 (11.386)	20.878 (9.075)	59.468 (12.119)	31.120 (6.757)	17.796 (11.180)	20.957 (6.296)
Mean length in Mb of ROH (SD)						
All (> 1.0 Mb)	4.385 (6.700)	4.821 (8.014)	7.980 (12.232)	5.452 (9.441)	5.073 (10.052)	5.098 (9.011)
Short (1.0 to 2.5 Mb)	1.602 (0.414)	1.576 (0.416)	1.635 (0.419)	1.569 (0.408)	1.602 (0.419)	1.581 (0.415)
Medium (2.5 to 5.0 Mb)	3.398 (0.678)	3.454 (0.695)	3.553 (0.728)	3.515 (0.717)	3.450 (0.699)	3.558 (0.706)
Long (> 5.0 Mb)	13.001 (10.956)	13.582 (12.874)	16.357 (15.816)	14.848 (14.948)	15.833 (17.956)	13.924 (14.746)

### Effective population size

**Fig. 6:**
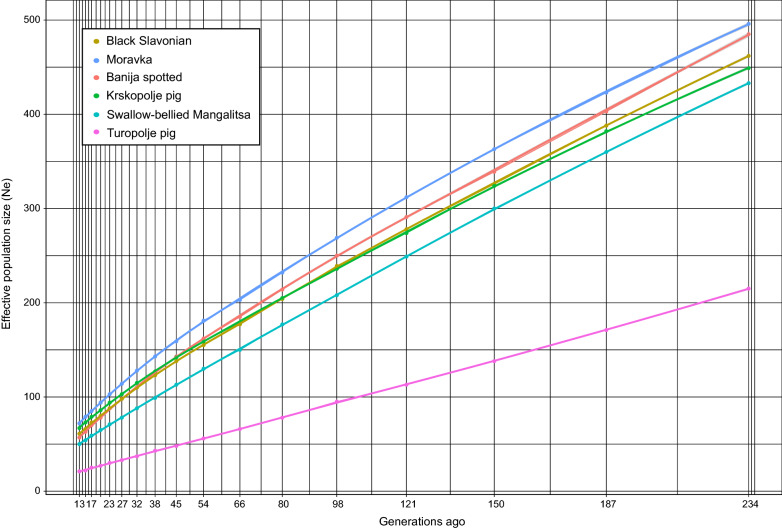
Trends in effective population size (${\mathrm{N}}_{\mathrm{eSNP}}$) over time (13 to 234 generations ago) estimated using genome-wide SNPs

The effective population size (${\mathrm{N}}_{\mathrm{e}}$) was estimated from pedigree (${\mathrm{N}}_{\mathrm{e PED}}$), microsatellite (${\mathrm{N}}_{\mathrm{e STR}}$) and SNP (${\mathrm{N}}_{\mathrm{e SNP}(13)}$) data, and ${\mathrm{N}}_{\mathrm{e}}$ was largest for the Black Slavonian breed, followed by the Banija spotted and Turopolje pig breeds. When considering only the microsatellite data, ${\mathrm{N}}_{\mathrm{e}}$ was largest for the Moravka and smallest for the Turopolje breeds. A similar ranking was observed when SNP data were used (see Additional file 5: Table S5). For the studied pig breeds, the effective population size tended to decline over time (Fig. 6). ${\mathrm{N}}_{\mathrm{e}}$ varied between the breeds, and 13 generations ago, it ranged from 21 to 72.

### Population structure and genetic distances

**Fig. 7:**
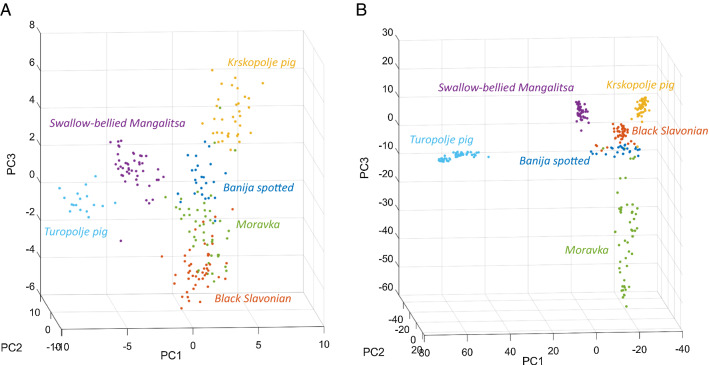
3D-PCA plots projecting genotyped samples using microsatellites (**a**) and SNPs (**b**)

Patterns of population structure assessed by PCA based on microsatellites and SNPs were similar (Fig. 7). With both types of markers, animals from different breeds were differentiated, but the clusters in the SNP-based PCA plot (b) were better defined than those in the microsatellite-based PCA plot (a).

**Fig. 8:**
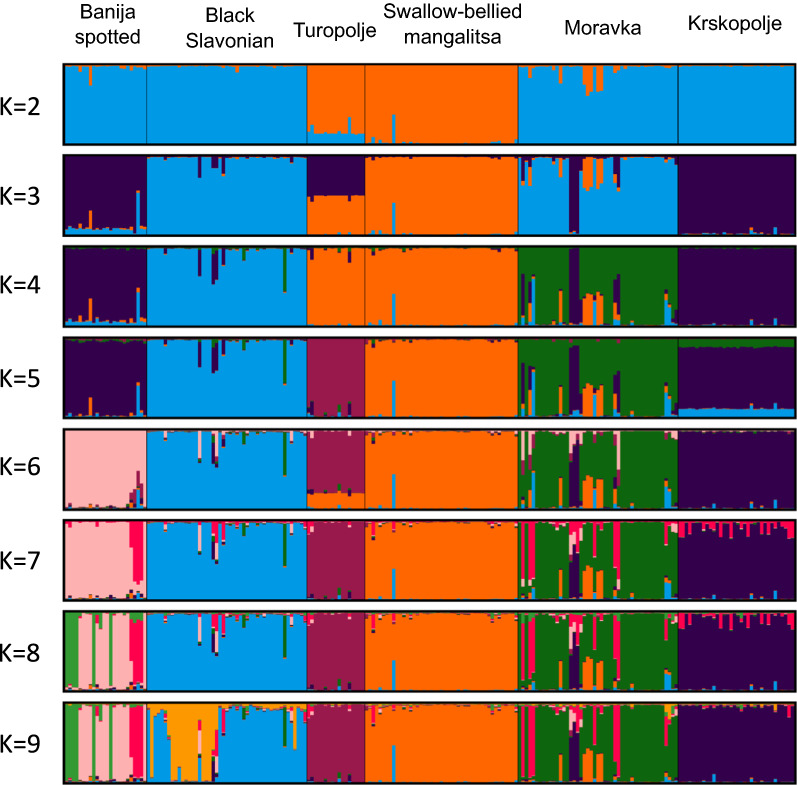
STRUCTURE analysis of the genetic structure of six autochthonous pig breeds based on microsatellite loci

**Fig. 9:**
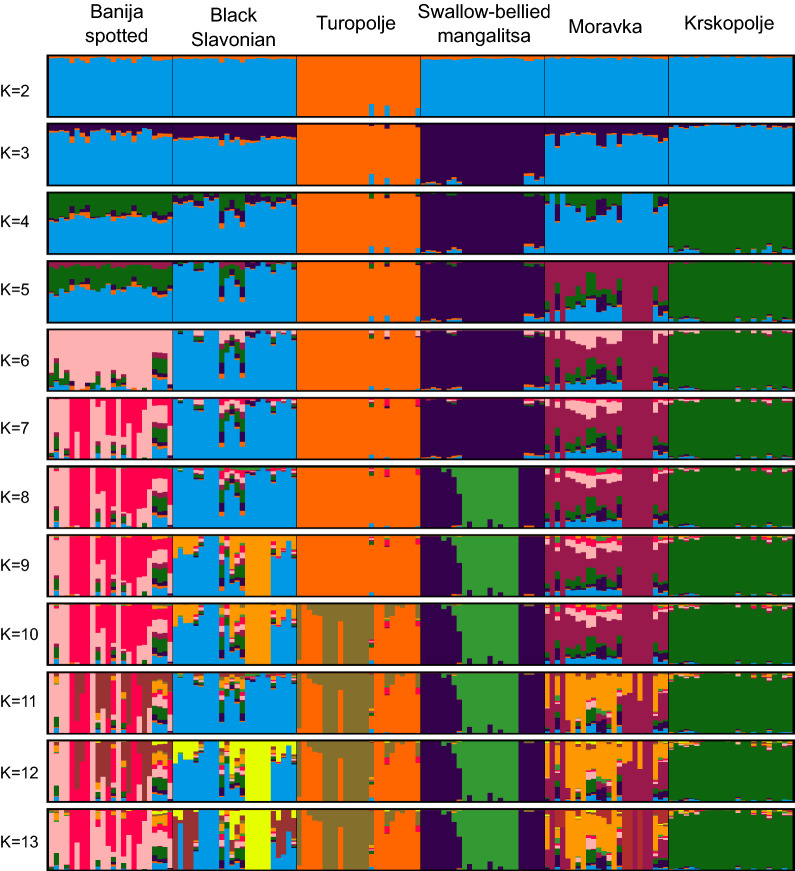
ADMIXTURE analysis of the genetic structure of six autochthonous pig breeds based on SNPs

Population structure was inferred using genetic clustering algorithms. STRUCTURE analysis was run on the microsatellite data (Fig. 8) and an optimal number of clusters of 7 was estimated. ADMIXTURE analysis was run on the pruned SNP data set (Fig. 9), and the lowest cross-validation error was estimated for 11 clusters (see Additional file 6: Fig. S1). A similar pattern of population differentiation across the K values was observed for both types of markers. At K = 2, microsatellites separate Turopolje and Swallow-bellied Mangalitsa from the other pig breeds, whereas SNPs separate only the Turopolje breed. The separation by the first principal component (PC1) is similar in the microsatellite- and SNP-based PCA (Fig. 7). At K = 6 (using microsatellites or SNPs), all analysed breeds are separated into relatively heterogeneous genetic clusters with some admixture traces, in particular for the Moravka breed.

### Genetic differentiation and spatial genetic structure

**Fig. 10:**
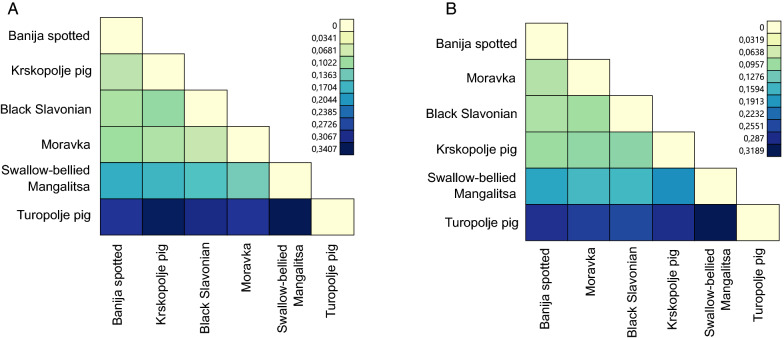
Nei’s genetic distances between the six autochthonous pig breeds based on **a** microsatellites and **b** SNPs

Pairwise ${\mathrm{F}}_{\mathrm{ST}}$ values indicate different genetic differentiation levels among the six analysed pig populations (Fig. 4) and (see Additional file 4: Table S4). Based on SNP data, the lowest observed ${\mathrm{F}}_{\mathrm{ST}}$ and the shortest Nei’s genetic distance were between the Moravka and Banija spotted breeds (${\mathrm{F}}_{\mathrm{ST SNP}}$= 0.085 and ${\mathrm{D}}_{\mathrm{SNP}}$ = 0.061), whereas based on microsatellite data, they were between the Moravka and Black Slavonian breeds (${\mathrm{F}}_{\mathrm{ST STR}}$ = 0.082 and ${\mathrm{D}}_{\mathrm{STR}}$ = 0.218) [see Fig. 10 and (see Additional file 7: Table S6)]. With both types of markers, ${\mathrm{F}}_{\mathrm{ST}}$ was highest for the Swallow-bellied Mangalitsa and Turopolje breeds (${\mathrm{F}}_{\mathrm{ST STR}}$ = 0.341 and ${\mathrm{F}}_{\mathrm{ST SNP}}$ = 0.341) and Nei’s genetic distance was greatest between the Krskopolje and Turopolje pig breeds (${\mathrm{D}}_{\mathrm{STR}}$ = 0.971 and ${\mathrm{D}}_{\mathrm{SNP}}$ = 0.163).

### Isolation-by-distance

**Fig. 11:**
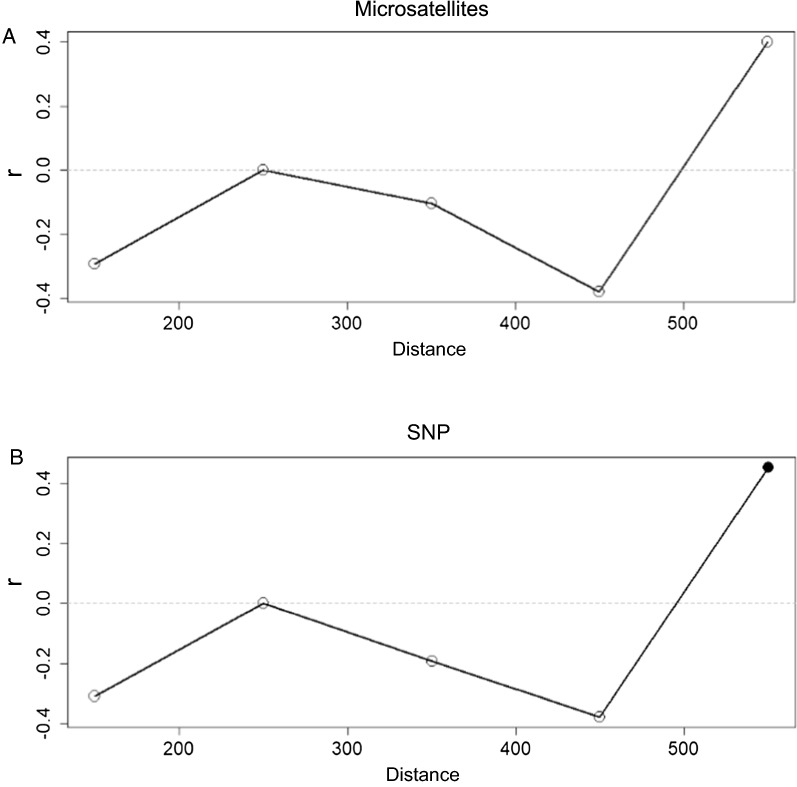
Mantel correlogram showing the structuring of local breeds within five classes of different distances based on **a** microsatellites and **b** SNPs. The black dot indicates a significant correlation

The Mantel test showed no significant correlation between genomic Nei’s distances and geographical distances for the six analysed pig breeds. Based on the microsatellite data, Nei’s and geographical distances indicate that there are no significant differences between populations caused by isolation-by-distance (${\mathrm{r}}_{\mathrm{m}}$ > 0.05 for all distance classes) (Fig. 11a). A similar pattern of correlation trend was observed on the correlograms based on SNP data and a significant correlation was observed for populations at distances greater than 550 km (${\mathrm{r}}_{\mathrm{m}}$ < 0.05) (Fig. 11b). The only two breeds for which the geographical distance was greater than 550 km in our data set were Krskopolje pig and Moravka.

### Genetic ancestry

The ancestral relationships between the six Balkan autochthonous pig breeds analysed here and pig populations worldwide (2113 individuals from 146 pig populations) were identified by calling shared haplotypes (identical-by-descent; IBD) (see Additional file 8: Fig. S2). The Turopolje pig breed showed the largest mean number of shared IBD haplotypes with the Banija spotted, Berkshire and Chinese Laiwuhei breeds and the wild boar. The Banija spotted breed shared IBD haplotypes with the Landrace and Bunte Bentheimer breeds, and the Krskopolje pig shared IBD haplotypes with the Angler Sattelschwein and Duroc breeds. The Black Slavonian breed showed the largest number of all size categories of shared segments with the Cinta Senese, Large Black and Berkshire breeds, and four or more of these segments were shared with the Swallow-bellied Mangalitsa and Chinese Jinhua breeds.

**Fig. 12:**
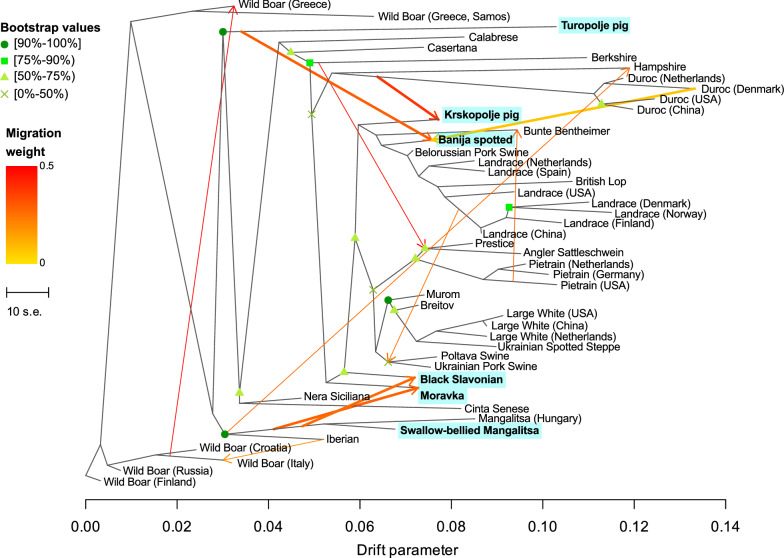
Maximum likelihood tree using the wild boar from Finland to root the tree. The arrows denote 11 migration events from the origin to the recipient breed. Migration arrows are coloured according to their weight

The 40 pig populations that showed the most extensive haplotype sharing with the six Balkan autochthonous pig breeds analysed in our study were selected for TreeMix analysis. The OptM function detected 11 migration events as the most reliable. This analysis also revealed possible introgressions from the Swallow-bellied Mangalitsa and Moravka breeds to the Black Slavonian breed and from Duroc to the Krskopolje pig and Banija spotted breeds. The TreeMix vector connected the ancestral population of the Turopolje pig with the Banija spotted breed (Fig. 12).

### Genomic patterns of homozygosity and signatures of selection

**Fig. 13:**
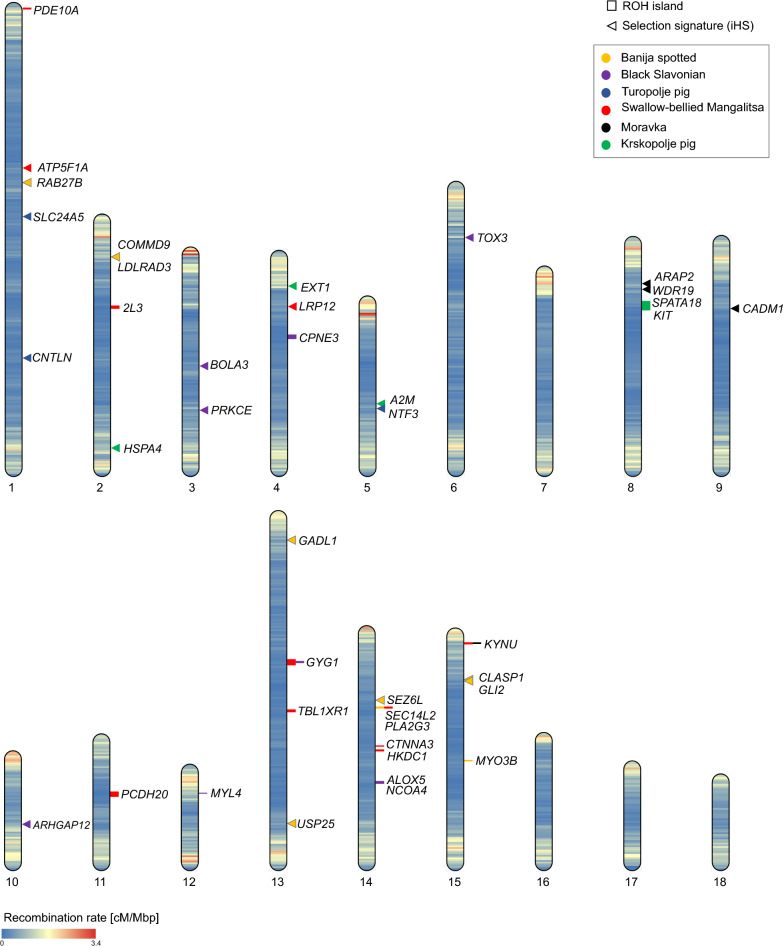
Chromosome idiograms showing the location of ROH islands and signatures of selection identified using iHS in the six autochthonous pig breeds. The colour of the horizontal lines within each chromosome presents the recombination rate according to the 21.5 Morgan map of the pig recombination landscape with the mean recombination rate of 0.95 cM/Mbp

Analysis of overlapping regions of extended homozygosity across breeds revealed unevenly distributed ROH islands on the autosomes (see Additional file 9: Fig. S3), which harbour QTL and genes associated with phenotypic traits in different mammalian species (see Additional file 10: Table S7). The highest percentage of animals that shared ROH was found in the Turopolje pig and Swallow-bellied Mangalitsa breeds. The ROH islands that were identified in the Swallow-bellied Mangalitsa on *Sus scrofa* chromosome (SSC) 13, 14 and 15, overlapped with ROH islands in Black Slavonian, Banija spotted and Moravka breeds, respectively (Fig. 13).

The integrated haplotype score (iHS) was used to identify within-breed signatures of selection (see Additional file 11: Fig. S4). Genes that were adjacent to the SNPs with the strongest iHS signals were identified and analysed for their putative roles in various biological pathways (see Additional file 12: Table S8). Candidate genes associated with female and male fertility were identified in several breeds: *ARHGAP12* [60] (Black Slavonian)*, ATP5F1A* [61] (Swallow-bellied Mangalitsa), *WDR19* [62] (Moravka) and *HSPA4* [63, 64] (Krskopolje pig). The *CNTL* [65] and *SLC24A5* [66] genes, which are involved in pigmentation, were detected in the Turopolje pig, whereas the *SEZ6L* [67] and *SLA-DRB* [68] genes that play a role in disease resistance, seemed to be under selection pressure in the Banija spotted and Black Slavonian breeds, respectively. Genes that are associated with pig production traits, such as *COMMD6* [69, 70], *LDLRAD3* [71], *USP25* [72], *GLI2* [73], *PRKCE* [74], *TOX3* [75], *LRP12* [76] and *EXT1* [77] have already been reported in the Alentejano, Casertana, Large White, Duroc and Iberian pig breeds. The gene *ARAP2* on SSC8 is associated with haematopoiesis in pig [78]. *GADL1*, *CLASP1* and *NTF3*, are candidate genes for *longissimus lumborum* muscle quality [79], Warner–Bratzler shear force [80] and cooking loss [81] in cattle, respectively. The *RAB27B* gene is associated with childhood body mass index [82]. However, in our data set we did not detect any co-localisation of ROH islands with signatures of selection using iHS (Fig. 13).

## Discussion

Our study focused on the genomic characterisation of six autochthonous Balkan pig breeds and addressed some challenges related to livestock genomic conservation [2]. The concept of genome conservation has been extensively discussed in the literature, but the advances in genomic technologies raise some new questions. With the transition from microsatellite to SNP data in recent years, we are faced with the problem of how to integrate data from both types of markers. Although microsatellites are sufficient for inferring genetic diversity and population structure, they are not sufficient for identifying polymorphisms that “cannot afford to be lost” from the local breeds.

### Rate of inbreeding and effective population size

We used two types of markers (microsatellites and SNPs) and also pedigree data, to estimate population parameters in the six autochthonous Balkan pig breeds analysed here, and we evaluated the consistency of these estimates. In practice, the availability of full and complete pedigree datasets is rare [83], and this was also confirmed in our study, with only little information available for all the pedigrees (Table 1). Consequently, based on pedigree data, inbreeding rates and coefficients may be underestimated and effective population sizes overestimated [84, 85]. Therefore, pedigree-derived estimates should be considered together with molecular data. Breeding programs in general, but especially programs for breeds under conservation, should include appropriate mating schemes to reduce the rate of inbreeding.

Estimates of effective population size (${\mathrm{N}}_{\mathrm{e}}$) derived from pedigree and molecular data showed low values for the six autochthonous Balkan pig breeds analysed (Fig. 6) (see Additional file 5: Table S5). The pedigree-based ${\mathrm{N}}_{\mathrm{e}}$ inferred for the three Croatian pig breeds ranged from 20.67 to 30.51 (see Additional file 5: Table S5), which is within the range that the Food and Agriculture Organization of United Nations (FAO, 2000) considers to classify breeds as endangered. However, the values for these six breeds are comparable to those for some other European indigenous breeds, such as Italian Mora Romagnola (10.87) and Cinta Senese (40.32), but are lower than those for commercial European pig breeds [86]. The results obtained using microsatellite and SNP data were similar; the Moravka breed had the largest ${\mathrm{N}}_{\mathrm{e}}$, while the Turopolje pig breed had the smallest ${\mathrm{N}}_{\mathrm{e}}$, which was expected since its current population size was the smallest (Croatian agency for food and agriculture, 2020) and it underwent a recent population bottleneck. According to the historical estimates based on SNP array data, the ${\mathrm{N}}_{\mathrm{e}}$ of all six breeds has decreased over the last 234 generations, with the Moravka breed showing the greatest decline (Fig. 6).

Estimates of inbreeding coefficient from pedigree and molecular data (Table 6) showed that the six autochthonous Balkan pig breeds analysed had a high level of inbreeding, which was expected due to their low ${\mathrm{N}}_{\mathrm{e}}$. However, relatively low levels of inbreeding were observed in the Banija spotted breed, which is the youngest among the analysed breeds and for which herdbook records began only in 2015. Such low inbreeding coefficients can be explained by controlled matings during the revitalisation process of the breed and by the small number of generations that separate the analysed individuals and the founders in the pedigree [14]. Inbreeding estimation based on genealogical data assumes the absence of genetic relatedness between founder animals in the pedigree, but this assumption can sometimes be violated, which biases the estimation of genealogical parameters [87].

Microsatellites have become one of the most widely used markers for estimating genetic variability and identifying individuals, mainly because of their high polymorphic nature and high informativeness [88, 89]. Based on the microsatellite data, the highest inbreeding coefficient was found for the Black Slavonian and Moravka breeds, but it was lower than that previously reported in 2019 by Gvozdanović et al. [13], who stated that the Black Slavonian breed population counted 1930 sows and 242 boars, which is less than the current population size of 2495 sows and 193 boars (Croatian agency for food and agriculture, 2020).

In recent years, the use of microsatellites for population genetic studies in livestock species has decreased due to the availability of SNP arrays and whole-genome sequencing (WGS) data. Due to their resolving power, lower rate of genotyping errors, reproducibility of genotyping, and high abundance in the genome of domestic animals, SNPs have become a tool of choice for various genomic analyses [90]. In our study, the highest ${\mathrm{F}}_{\mathrm{IS}}$ value estimated from SNP data (Table 6) was observed for the Moravka breed (0.021) and the lowest for the Turopolje breed (-0.039), the latter indicating an excess of heterozygosity.

The coefficient of inbreeding derived from ROH (${\mathrm{F}}_{\mathrm{ROH}}$) can provide a more accurate measure of inbreeding level compared to that based on pedigree records [91]. Our results show that ${\mathrm{F}}_{\mathrm{ROH}}$ does not differ significantly among the analysed breeds, except for the Turopolje pig breed with the highest ${\mathrm{F}}_{\mathrm{ROH}}$ value (0.508) (Table 6). This breed also had the highest ${\mathrm{F}}_{\mathrm{PED}}$ value (0.038), which results from its small population size and frequent uncontrolled mating as well as from difficulties in implementing the breeding program for this breed [92]. The ${\mathrm{F}}_{\mathrm{ROH}}$ coefficients for the other five breeds in our study were comparable to those for other European local pig breeds such as Cinta Senese (0.147) and Casertana (0.226) but higher than those for commercial pig breeds (Large White, 0.075) [93]. On the contrary, the average ${\mathrm{F}}_{\mathrm{ROH}}$ values (0.14) reported by Bâlteanu et al. [94] for the Swallow-bellied Mangalitsa breed was lower than that obtained in our study. Such a difference may be the result of heavy and recent inbreeding, probably due to severe reductions in the census. Our estimated ${\mathrm{F}}_{\mathrm{ROH}}$ coefficients are also higher than those recently published by Schiavo et al. [93] for five Balkan breeds, which might be explained by the different definition of ROH used in the two studies.

Mean lengths and number of ROH varied among the six autochthonous Balkan pig breeds analysed, but the mean number of ROH per population increased with increasing mean length of ROH (Table 7). Short ROH have evolved in the past as a result of ancestral recombination processes [95]. The largest mean number of short ROH was observed in the Swallow-bellied Mangalitsa breed, followed by the Turopolje pig, which indicates an earlier occurrence of inbreeding. This is supported by evidence of intentional matings between closely-related Turopolje pig individuals, which seems to have been a common breeding practice in the history of this breed [92]. The presence of long ROH and their larger number indicate recent inbreeding events [96], which can also be observed in the six analysed pig breeds with the largest number of long ROH detected in the Turopolje and the Swallow-bellied Mangalitsa pig breeds.

### Genetic variability

Our results show that the genetic variability parameters calculated using microsatellite and SNP data were similar (Table 5). Estimates of heterozygosity (${\mathrm{H}}_{\mathrm{exp}}$ and ${\mathrm{H}}_{\mathrm{obs}}$) using both types of markers were consistent although those based on microsatellites were higher than those based on SNP data, which can be explained by the highly polymorphic nature of microsatellites and the limited amount and ascertainment bias of SNP data [97]. The lowest heterozygosity values calculated using microsatellite and SNP data were found for the Turopolje pig, which agrees with previous studies based on microsatellites [13, 19], and indicates that a smaller number of boars has been used in its mating systems, as shown in [98]. The highest microsatellite-based ${\mathrm{H}}_{\mathrm{exp}}$ was found for the Krskopolje pig and is in agreement with a previous study by Flisar et al. [18]. The highest SNP-based ${\mathrm{H}}_{\mathrm{exp}}$ was found for the Banija spotted breed. A microsatellite-based analysis for this breed was performed by Šalamon et al. [12] who reported ${\mathrm{H}}_{\mathrm{obs}}$ and ${\mathrm{H}}_{\mathrm{exp}}$ values of 0.58 and 0.61, respectively. They also noted that this breed has a high level of genetic diversity and is clearly differentiated from other geographically-related pig populations.

The genetic differentiation between the populations was assessed by ${\mathrm{F}}_{\mathrm{ST}}$ (Table 5) with the highest value obtained for the Moravka breed (0.185), which indicates that 18.5% of the genetic variation in the Moravka breed is explained by differences between the studied populations and the remaining 81.5% is due to differences between individuals. The Turopolje pig had the lowest ${\mathrm{F}}_{\mathrm{ST}}$ value (0.123), and also the lowest overall inbreeding coefficient of an individual compared to the total population (${\mathrm{F}}_{\mathrm{IT}}$ = 0.155), which indicates a low level of genetic differentiation of the individuals compared to the total population. The average number of alleles varied from 3.125 in the Turopolje to 6.542 in the Moravka breed, which is consistent with the criterion of Barker [99], who suggested that microsatellite markers, used to estimate genetic distances, should have at least four alleles to reduce the standard errors of these estimates.

### Population structure and genetic differentiation

Population structure and genetic differentiation were assessed using microsatellite and SNP data for principal component analysis (PCA), unsupervised Bayesian clustering algorithms (STRUCTURE and ADMIXTURE), population pairwise ${\mathrm{F}}_{\mathrm{ST}}$, Nei’s genetic distances, and isolation-by-distance analysis. Using microsatellite or SNP data produced a similar pattern of breed differentiation. In the PCA plot based on both types of markers (Fig. 7), Turopolje pig and Swallow-belied Mangalitsa are separated from the other breeds by the first principal component (PC1). The PCA results are supported by pairwise F_ST_ values between breeds (Fig. 4) and the unsupervised clustering analysis (Figs. 8 and 9). Although the formation of the Banija spotted breed was influenced by the Turopolje pig, they form two separate clusters. Considering that Turopolje pig is one of the oldest pig breeds in Europe, its early differentiation from Banija spotted and from other pig breeds is expected. According to the microsatellite-based analysis, the Banija spotted breed clustered together with the Krskopolje pig, which contrasts with the SNP-based results that show that the Banija spotted breed clustered together with the Black Slavonian breed. Previous results, based on microsatellites, showed little differentiation between these two breeds [12]. However, both marker types and both approaches (PCA and unsupervised clustering) successfully separated the six breeds analysed here, and the animals that belonged to the same breed formed relatively compact clusters. Modelling for a number of source populations larger than six revealed some potential subclusters within populations; the Black Slavonian breed might have two (according to the microsatellite-based analysis) (Fig. 8) or three (according to the SNP-based analysis) (Fig. 9) subpopulations, which is consistent with the results of Gvozdanović et al. [13] who performed a microsatellite-based analysis that suggested the existence of three genetic pools within the Black Slavonian population as a consequence of uncontrolled crossing with modern pig breeds (Duroc). Most of the autochthonous Balkan pig breeds have been crossed with other European pig breeds during their history, resulting in indirect introgression of the Asian gene pool [4, 100].

In this study, we tested the relevance of the isolation-by-distance model to describe the genetic differentiation between the analysed breeds (Fig. 11). The concept of isolation-by-distance was introduced by Wright [101] and describes the correlation between geographical and genetic distances, which is the consequence of a limited dispersal of genetic material over geographical areas. The correlations between the matrices of geographical and genetic distances among the breeds in our study showed that isolation-by-distance was not significant for most distance classes. Because the breeds in our study inhabit a relatively small geographic area and share a common historical context, it is reasonable to assume that some significant migration of genetic material occurred from sources that participated in the formation of the breeds, which resulted in low correlations between geographic and genetic distances among the breeds studied.

Our results are consistent with those of Traspov et al. [102], which showed that pig breeds in Eastern Europe displayed no geographical structure, and also with the study of Yang et al. [26], who found no correlation between genetic and geographical distances in European pig breeds. This lack of correlation is attributed to the introgression of Asian breeds and the intensive use of highly productive cosmopolitan pig breeds, both of which influenced local pig populations.

The two marker panels used in our analysis were sufficiently informative to differentiate between the six autochthonous Balkan pig breeds analysed here. Regarding the differentiation between populations, random microsatellites were more informative than random SNPs (Fig. 3). However, when a sufficient number of SNPs was genotyped, the inference on population structure was better than that based on microsatellites (Fig. 7). We calculated Rosenberg’s informativeness ${\mathrm{I}}_{\mathrm{n}}$ for inference of population structure [45], and the ratio of the mean informativeness for microsatellites (mean ${\mathrm{I}}_{\mathrm{n STR}}$ = 0.45) to that for SNPs (mean ${\mathrm{I}}_{\mathrm{n SNP}}$_=_ 0.083) was 5.42, which is consistent with results observed in other studies [45, 103].

### Genetic ancestry of the analysed pig breeds

TreeMix analysis (Fig. 12) was performed to investigate potential admixture between the six analysed pig breeds and with commercial and autochthonous pig populations. Moderate gene flow was observed between the Swallow-bellied Mangalitsa and Moravka breeds and between the Swallow-bellied Mangalitsa and Black Slavonian breeds. Introgression from the Swallow-bellied Mangalitsa to the Moravka breed could be due to crossbreeding events that occurred in the near past to improve production traits of the Mangalitsa breed [104]; the link between the Swallow-bellied Mangalitsa and Black Slavonian breeds is not surprising, since the formation of the Black Slavonian breed was based on the crossbreeding of the Swallow-bellied Mangalitsa with other pig breeds (Berkshire, Poland China and Large Black pig) [5]. Moreover, Ribani et al. [105] found that the frequency of the *MC1R* allele *E*^*D1*^ is high in both the Black Slavonian and Swallow-bellied Mangalitsa breeds (88 and 37%, respectively), which suggests a common genetic origin. We also observed a genetic contribution of the Duroc breed to the Banija spotted and Krskopolje pig breeds. Crossing of autochthonous pig breeds with conventional breeds such as Duroc has often been used to improve low reproduction parameters [106].

### Genomic regions under selection pressure

The distribution of ROH across the genome is population-specific [107]. Identification of ROH islands is considered an effective method to identify genomic regions under natural or artificial selection [108]. ROH islands were detected in the six breeds analysed here (see Fig. 13, and (see Additional file 8: Fig. S2 and Additional file 9: Fig. S3]). Several genomic regions show an extremely high frequency of ROH in the Swallow-bellied Mangalitsa breed. The ROH island that is located on SSC13 and harbours the *PLSCR4, GYG1* and *HPS3* genes is shared by 98% of the genotyped individuals in this breed. These three genes are associated with total number born and number born alive in pigs (*PLSCR4*) [109], glycogen metabolism in cattle (*GYG1*) [110] and brown coat colour in dogs (*HPS3*) [111]. However, a part of this ROH island was also observed in the Black Slavonian breed (Fig. 13) and is shared by 73% of the animals. Another ROH island that is shared by 98% of the Swallow-bellied Mangalitsa animals is positioned on SSC14 and harbours more than 20 genes, among which are *SEC14L2* that is associated with the regulation of cholesterol biosynthesis in mice [112], and *PLA2G3* associated with fatty acid metabolism [113]. A non-synonymous SNP, specific to the Swallow-bellied Mangalitsa breed, was observed in the *PLA2G3* gene [114]. This ROH island on SSC14 was also observed in 75% of the Banija spotted and Large White pigs [114]. The ROH island on SSC15 that is present in 70% of the Moravka pigs is also shared with the Swallow-bellied Mangalitsa breed and contains the *KYNU* gene, which is associated with daily weight gain in Duroc boars [115] and is involved in the kynurenine pathway, i.e. the main pathway for tryptophan metabolism [116]. Finally, the ROH island on SSC8 detected in the Krskopolje pig harbours the *KIT* gene, which is associated with coat colour and the white belted phenotype that characterises this breed [117, 118].

In addition, another approach was used to detect genomic traces of selective events within the analysed breeds. The iHS method compares extended haplotype homozygosity (EHH) [119] between derived and ancestral alleles within populations and can detect ongoing selection processes where the target allele has a moderate to high frequency within a population. Using the iHS method, several candidate genes for reproduction traits and disease resistance were discovered (see Additional file 11: Fig. S4 and Additional file 12: Table S8).

## Conclusions

We analysed six indigenous pig breeds from Croatia, Serbia, and Slovenia using two types of molecular markers (microsatellites and SNPs) and pedigree data. Genetic diversity estimates for both types of markers were generally consistent but some of the observed differences can be explained by the highly polymorphic nature of microsatellites and by the limited amount and ascertainment bias of SNP data [97]. The lowest heterozygosity values calculated from microsatellite and SNP data were found for the Turopolje pig, and ${\mathrm{H}}_{\mathrm{obs}}$ was higher than ${\mathrm{H}}_{\mathrm{exp}}$ for the Black Slavonian, Turopolje pig and Moravka breeds. Both types of markers allowed to distinguish clusters of individuals belonging to each breed. The analysis of potential admixture of the analysed pig breeds with commercial and other autochthonous breeds as well as wild boar revealed potential gene flow between the Mangalitsa and Moravka breeds and between the Mangalitsa and Black Slavonian pig breeds.

The distribution of ROH across the genome was not uniform. ROH island analysis revealed genomic regions with an extremely high frequency of shared ROH in the Mangalitsa breed, which harbour the *SEC14L2*, *PLA2G3* and *KYNU* genes that are associated with cholesterol biosynthesis, fatty acid metabolism and daily weight gain, respectively. The iHS approach that detects signatures of selection revealed candidate regions containing genes with a potential role in reproduction traits; *ARHGAP12* (in Black Slavonian), *ATP5F1A* (in Swallow-bellied Mangalitsa), *WDR19* (in Moravka), *HSPA4* and *A2M* (in Krskopolje pig). In addition, the *SEZ6L* and *SLA-DRB* genes associated with disease resistance were identified in regions under selection pressure in the Banija spotted and Black Slavonian breeds.

## Supplementary Information

**Additional file 1: Table S1.** Primer sequences. Three multiplex reactions with microsatellite markers, corresponding chromosome location, primer sequence with an indication of the dye used for labelling, annealing temperature and fragment size.

**Additional file 2: Table S2.** Statistics of genetic diversity across the microsatellites used in this study. Statistics of the genetic diversity across the 24 microsatellites used in the Banija spotted, Black Slavonian, Turopolje pig, Swallow-bellied Mangalitsa, Moravka and Krskopolje pig breeds.

**Additional file 3: Table S3.** Pairwise ${\mathrm{F}}_{\mathrm{ST}}$ based on microsatellite and SNP markers.

**Additional file 4: Table S4.** List of the most informative SNPs.

**Additional file 5: Table S5.** Effective population size (${\mathrm{N}}_{\mathrm{e}}$).

**Additional file 6: Figure S1.** Optimal number of genetic clusters. Evanno’s statistic ΔK, showing a peak at the optimal number of clusters (A) and cross-validation (CV) error estimates of ADMIXTURE, with varying levels of K (B).

**Additional file 7: Table S6.** Nei's genetic distances based on microsatellites and SNPs.

**Additional file 8: Figure S2.** Breed-pairwise shared identical-by-descent segments (> 7 Mb, 3-7 Mb, < 3Mbp).

**Additional file 9: Figure S3.** Manhattan plot of ROH islands.

**Additional file 10: Table S7.** ROH islands in the Banija spotted, Black Slavonian, Swallow-bellied Mangalitsa, Moravka and Krskopolje pig breeds [109–115, 117, 118, 120–136].

**Additional file 11: Figure S4.** Manhattan plot of signatures of selection (iHS).

**Additional file 12: Table S8.** Signatures of selection in the Banija spotted, Black Slavonian, Turopolje pig, Swallow-bellied Mangalitsa, Moravka and Krskopolje pig breeds [60–82, 137–140].

## Data Availability

The data are available from the authors upon request.
