# Supplementary material for: Genetic diversity and population structure of six autochthonous pig breeds from Croatia, Serbia, and Slovenia
Source: Genet Sel Evol. 2022 Apr 28;54:30. doi: 10.1186/s12711-022-00718-6 (PMC9052598; doi:10.1186/s12711-022-00718-6)
Supplement: Supplementary file 1 — Additional file 1: Table S1. Primer sequences. Three multiplex reactions with microsatellite markers, corresponding chromosome location, primer sequence with an indication of the dye used for labelling, annealing temperature and fragment size. [file 12711_2022_718_MOESM1_ESM.docx]

Table S1

Three multiplex reactions with microsatellite markers, corresponding chromosome location, primer sequence with an indication of dye used for labelling, annealing temperature and fragment size

| **Marker name** | **Multiplex group** | **Chr.** | **Primer sequence (5’ -> 3’)** | **Temperature** | **Fragment size/bp** | **Fluorescent label ('5')** |
| --- | --- | --- | --- | --- | --- | --- |
| S0026 | 1 | 16 | AACCTTCCCTTCCCAATCAC CACAGACTGCTTTTTACTCC | 55°C | 87 - 105 | FAM |
| S0155 | 1 | 1 | TGTTCTCTGTTTCTCCTCTGTTTG AAAGTGGAAAGAGTCAATGGCTAT | 55°C | 142 - 162 | FAM |
| S0005 | 1 | 5 | TCCTTCCCTCCTGGTAACTA GCACTTCCTGATTCTGGGTA | 55°C | 203 - 267 | FAM |
| Sw2410 | 1 | 8 | ATTTGCCCCCAAGGTATTTC CAGGGTGTGGAGGGTAGAAG | 50°C | 90 - 131 | ATTO |
| Sw830 | 1 | 10 | AAGTACCATGGAGAGGGAAATG ACATGGTTCCAAAGACCTGTG | 50°C | 168 - 203 | ATTO |
| S0355 | 1 | 15 | TCTGGCTCCTACACTCCTTCTTGATG TTGGGTGGGTGCTGAAAAATAGGA | 50°C | 244 - 271 | ATTO |
| Sw24 | 1 | 17 | CTTTGGGTGGAGTGTGTGC ATCCAAATGCTGCAAGCG | 55°C | 95 - 124 | HEX |
| Sw632 | 1 | 7 | TGGGTTGAAAGATTTCCCAA GGAGTCAGTACTTTGGCTTGA | 55°C | 148 - 178 | HEX |
| Sw1941 | 1 | 13 | AGAAAGCAATTTGATTTGCATAATC ACAAGGACCTACTGTATAGCACAGG | 55°C | 202 - 224 | HEX |
| Sw9366 | 2 | 15 | TCTGGAGCTAGCATAAGTGCC  GTGCAAGTACACATGCAGGG | 55°C | 90 - 116 | FAM |
| S0218 | 2 | X | GTGTAGGCTGGCGGTTGT CCCTGAAACCTAAAGCAAAG | 55°C | 158 - 205 | FAM |
| S0228 | 2 | 6 | GGCATAGGCTGGCAGCAACA AGCCCACCTCATCTTATCTACACT | 55°C | 220 - 246 | FAM |
| Sw122 | 2 | 6 | CAAAAAAGGCAAAAGATTGACA TTGTCTTTTTATTTTGCTTTTGG | 55°C | 106 - 128 | ATTO |
| Sw857 | 2 | 14 | TGAGAGGTCAGTTACAGAAGACC GATCCTCCTCCAAATCCCAT | 55°C | 141 - 159 | ATTO |
| S0097 | 2 | 4 | GACCTATCTAATGTCATTATAGT TTCCTCCTAGAGTTGACAAACTT | 55°C | 209 - 250 | ATTO |
| sw240 | 2 | 2 | AGAAATTAGTGCCTCAAATTGG AAACCATTAAGTCCCTAGCAAA | 55°C | 92 - 124 | HEX |
| Sw2406 | 2 | 6 | AATGTCACCTTTAAGACGTGGG AATGCGAAACTCCTGAATTAGC | 55°C | 222 - 262 | HEX |
| Sw72 | 3 | 3 | ATCAGAACAGTGCGCCGT TTTGAAAATGGGGTGTTTCC | 55°C | 97 - 114 | FAM |
| S0226 | 3 | 2 | GCACTTTTAACTTTCATGATACTCC GGTTAAACTTTTNCCCCAATAC | 55°C | 180 - 210 | FAM |
| S0090 | 3 | 12 | CCAAGACTGCCTTGTAGGTGAATA  GCTATCAAGTATTGTACCATTAGG | 55°C | 227 - 249 | FAM |
| Sw2008 | 3 | 11 | CAGGCCAGAGTAGCGTGC  CAGTCCTCCCAAAAATAACATG | 55°C | 95 - 108 | ATTO |
| Sw1067 | 3 | 6 | TGCTGGCCAGTGACTCTG CCGGGGGATTAAACAAAAAG | 55°C | 136 - 176 | ATTO |
| S0101 | 3 | 7 | GAATGCAAAGAGTTCAGTGTAGG GTCTCCCTCACACTTACCGCAG | 58°C | 197 - 221 | ATTO |
| Sw911 | 3 | 9 | CTCAGTTCTTTGGGACTGAACC CATCTGTGGAAAAAAAAAGCC | 60°C | 149 - 173 | HEX |
| S0002 | 3 | 3 | GAAGCCAAAGAGACAACTGC  GTTCTTTACCCACTGAGCCA | 60°C | 186 - 216 | HEX |
