# Supplementary material for: Genetic diversity and population structure of six autochthonous pig breeds from Croatia, Serbia, and Slovenia
Source: Genet Sel Evol. 2022 Apr 28;54:30. doi: 10.1186/s12711-022-00718-6 (PMC9052598; doi:10.1186/s12711-022-00718-6)
Supplement: Supplementary file 2 — Additional file 2: Table S2. Statistics of genetic diversity across the microsatellites used in this study. Statistics of the genetic diversity across the 24 microsatellites used in the Banija spotted, Black Slavonian, Turopolje pig, Swallow-bellied Mangalitsa, Moravka and Krskopolje pig breeds. [file 12711_2022_718_MOESM2_ESM.docx]

**Table S2**

Statistics of genetic diversity across 24 STR loci in Banija spotted, Black Slavonian, Turopolje, Swallow-bellied Mangalitsa, Moravka and Krskopolje pig breeds

| **Locus** | **Na** | **In** | **1-D** | **H** | **GD** | **Evenness** | **PIC** |
| --- | --- | --- | --- | --- | --- | --- | --- |
| S0005 | 18 | 0.961 | 0.91 | 2.55 | 0.91 | 0.82 | 0.899 |
| S0101 | 10 | 0.595 | 0.77 | 1.72 | 0.77 | 0.71 | 0.739 |
| S0002 | 13 | 0.590 | 0.81 | 1.93 | 0.81 | 0.72 | 0.787 |
| S0226 | 8 | 0.563 | 0.80 | 1.76 | 0.80 | 0.81 | 0.589 |
| Sw9366 | 10 | 0.555 | 0.82 | 1.88 | 0.82 | 0.81 | 0.797 |
| Sw1067 | 14 | 0.553 | 0.82 | 1.90 | 0.82 | 0.79 | 0.795 |
| Sw830 | 11 | 0.530 | 0.84 | 2.03 | 0.85 | 0.82 | 0.827 |
| S0355 | 10 | 0.504 | 0.79 | 1.73 | 0.79 | 0.82 | 0.760 |
| Sw2410 | 13 | 0.491 | 0.80 | 1.84 | 0.80 | 0.75 | 0.774 |
| Sw911 | 8 | 0.476 | 0.77 | 1.57 | 0.77 | 0.89 | 0.738 |
| Sw122 | 9 | 0.456 | 0.85 | 2.00 | 0.85 | 0.90 | 0.836 |
| Sw857 | 7 | 0.446 | 0.76 | 1.63 | 0.77 | 0.79 | 0.732 |
| S0097 | 11 | 0.418 | 0.82 | 1.92 | 0.82 | 0.78 | 0.798 |
| Sw2406 | 9 | 0.414 | 0.71 | 1.54 | 0.71 | 0.68 | 0.681 |
| S0155 | 8 | 0.401 | 0.77 | 1.61 | 0.77 | 0.85 | 0.735 |
| Sw632 | 8 | 0.373 | 0.55 | 1.24 | 0.55 | 0.50 | 0.528 |
| sw240 | 11 | 0.365 | 0.82 | 1.88 | 0.82 | 0.79 | 0.794 |
| SO090 | 11 | 0.343 | 0.74 | 1.57 | 0.74 | 0.73 | 0.702 |
| Sw24 | 11 | 0.338 | 0.59 | 1.31 | 0.59 | 0.53 | 0.560 |
| Swr1941 | 8 | 0.335 | 0.47 | 0.98 | 0.47 | 0.53 | 0.437 |
| S0218 | 8 | 0.317 | 0.51 | 0.97 | 0.51 | 0.62 | 0.448 |
| S0026 | 6 | 0.307 | 0.63 | 1.26 | 0.63 | 0.66 | 0.773 |
| Sw72 | 7 | 0.306 | 0.72 | 1.45 | 0.72 | 0.77 | 0.672 |
| S0228 | 6 | 0.183 | 0.42 | 0.88 | 0.43 | 0.53 | 0.399 |

Na – Number of alleles observed, In – Informativeness, 1-D – Simpson’s index, H – Shannon-Wiener index, GD – Nei’s 1978 unbiased gene diversity, PIC - polymorphic information content
