# Supplementary material for: Genetic diversity and population structure of six autochthonous pig breeds from Croatia, Serbia, and Slovenia
Source: Genet Sel Evol. 2022 Apr 28;54:30. doi: 10.1186/s12711-022-00718-6 (PMC9052598; doi:10.1186/s12711-022-00718-6)
Supplement: Supplementary file 3 — Additional file 3: Table S3. Pairwise ${\mathrm{F}}_{\mathrm{ST}}$FST based on microsatellite and SNP markers. [file 12711_2022_718_MOESM3_ESM.docx]

**Table S3**

SNPs with the highest informativeness value (I_n_ = 0.45)

| **Marker name** | **rs ID** | **Genomic position** | **Genomic context** |
| --- | --- | --- | --- |
| Ssc9_MT_4197* | / | / | / |
| WU_10.2_10_92752 | rs322882748 | 3:22661316 | *EARS2* (intron variant) |
| WU_10.2_4_805454 | rs333310144 | 4:706610 | *PLEC* (intron variant) |
| WU_10.2_4_115323970 | rs339635027 | 4:105284193 | *NGF* (intron variant) |
| WU_10.2_5_53024954 | rs342609364 | 5:49361377 | *SOX5* (intron variant) |
| WU_10.2_6_11354168 | rs335106691 | 6:11584404 | *CNTNAP4* (intron variant) |
| WU_10.2_7_16914276 | rs343783270 | 7:16004224 | *CDKAL1* (intron variant) |
| WU_10.2_7_129215847* | rs345177375 | / | / |
| ALGA0102731 | rs81329601 | 8:41371756 | intergenic variant |
| WU_10.2_11_5615159 | rs321295377 | 11:5926028 | *ENSSSCG00000049125* (ncRNA) |
| WU_10.2_11_8438844* | rs336939096 | / | / |
| WU_10.2_11_79476908 | rs328065019 | 11:72058840 | intergenic variant |
| WU_10.2_12_4286770 | rs341638370 | 12:4539508 | *ENSSSCG00000033293* (ncRNA) |
| WU_10.2_14_11870413 | rs345563351 | 14:10693501 | *ADRA1A* (intron variant) |
| 19_126183793* | / | / | / |

* Variants do not map to the reference genome (Ensembl 102, Sscrofa11.1).
