# Supplementary material for: Genetic diversity and population structure of six autochthonous pig breeds from Croatia, Serbia, and Slovenia
Source: Genet Sel Evol. 2022 Apr 28;54:30. doi: 10.1186/s12711-022-00718-6 (PMC9052598; doi:10.1186/s12711-022-00718-6)
Supplement: Supplementary file 4 — Additional file 4: Table S4. List of the most informative SNPs. [file 12711_2022_718_MOESM4_ESM.docx]

**Table S4**

Pairwise F_ST_ based on STR and SNP markers

|  | **Banija spotted** | **Black Slavonian** | **Krskopolje** | **Swallow-bellied Mangalitsa** | **Moravka** | **Turopolje** |
| --- | --- | --- | --- | --- | --- | --- |
| **STR** | | | | | | |
| **Banija Spotted** | 0.000 | - | - | - | - | - |
| **Black Slavonian** | 0.095 | 0.000 | - | - | - | - |
| **Krskopolje** | 0.088 | 0.106 | 0.000 | - | - | - |
| **Swallow-bellied Mangalitsa** | 0.180 | 0.158 | 0.169 | 0.000 | - | - |
| **Moravka** | 0.099 | 0.082 | 0.093 | 0.132 | 0.000 | - |
| **Turopolje** | 0.295 | 0.308 | 0.335 | 0.341 | 0.294 | 0.000 |
| **SNP** | | | | | | |
| **Banija spotted** | 0.000 | - | - | - | - | - |
| **Black Slavonian** | 0.098 | 0.000 | - | - | - | - |
| **Krskopolje** | 0.094 | 0.118 | 0.000 | - | - | - |
| **Swallow-bellied Mangalitsa** | 0.174 | 0.171 | 0.197 | 0.000 | - | - |
| **Moravka** | 0.085 | 0.104 | 0.102 | 0.156 | 0.000 | - |
| **Turopolje** | 0.304 | 0.309 | 0.305 | 0.341 | 0.288 | 0.000 |
