# Supplementary material for: Genetic diversity and population structure of six autochthonous pig breeds from Croatia, Serbia, and Slovenia
Source: Genet Sel Evol. 2022 Apr 28;54:30. doi: 10.1186/s12711-022-00718-6 (PMC9052598; doi:10.1186/s12711-022-00718-6)
Supplement: Supplementary file 5 — Additional file 5: Table S5. Effective population size (${\mathrm{N}}_{\mathrm{e}}$Ne). [file 12711_2022_718_MOESM5_ESM.docx]

**Table S5**

Effective population size (N_e_)

| **Breed** | **N_ePED_** | **N_eSTR_** | **N_eSNP_** | | | |
| --- | --- | --- | --- | --- | --- | --- |
|  |  |  | 13 gen. ago | 54 gen. ago | 98 gen. ago | 234 gen. ago |
| Banija spotted | 28.81 | 13.0 | 57 | 162 | 250 | 485 |
| Black Slavonian | 30.51 | 25.3 | 61 | 155 | 239 | 462 |
| Turopolje pig | 20.67 | 7.6 | 21 | 56 | 95 | 215 |
| Swallow-bellied Mangalitsa | - | 20.1 | 50 | 130 | 208 | 433 |
| Moravka pig | - | 26.8 | 72 | 181 | 269 | 496 |
| Krskopolje pig | - | 18.3 | 67 | 159 | 236 | 449 |

Estimates of effective population size: N_ePED_ – based on pedigree data, N_eSTR_ - based on STR markers, N_eSNP_ – based on SNP data, gen. ago – generations ago
