# Supplementary figures and images for: Genetic diversity and population structure of six autochthonous pig breeds from Croatia, Serbia, and Slovenia
Source: Genet Sel Evol. 2022 Apr 28;54:30. doi: 10.1186/s12711-022-00718-6 (PMC9052598; doi:10.1186/s12711-022-00718-6)

A)

$$\text{Delta K} = \text{mean}(|L''(K)|) / \text{stdev}[L(K)]$$

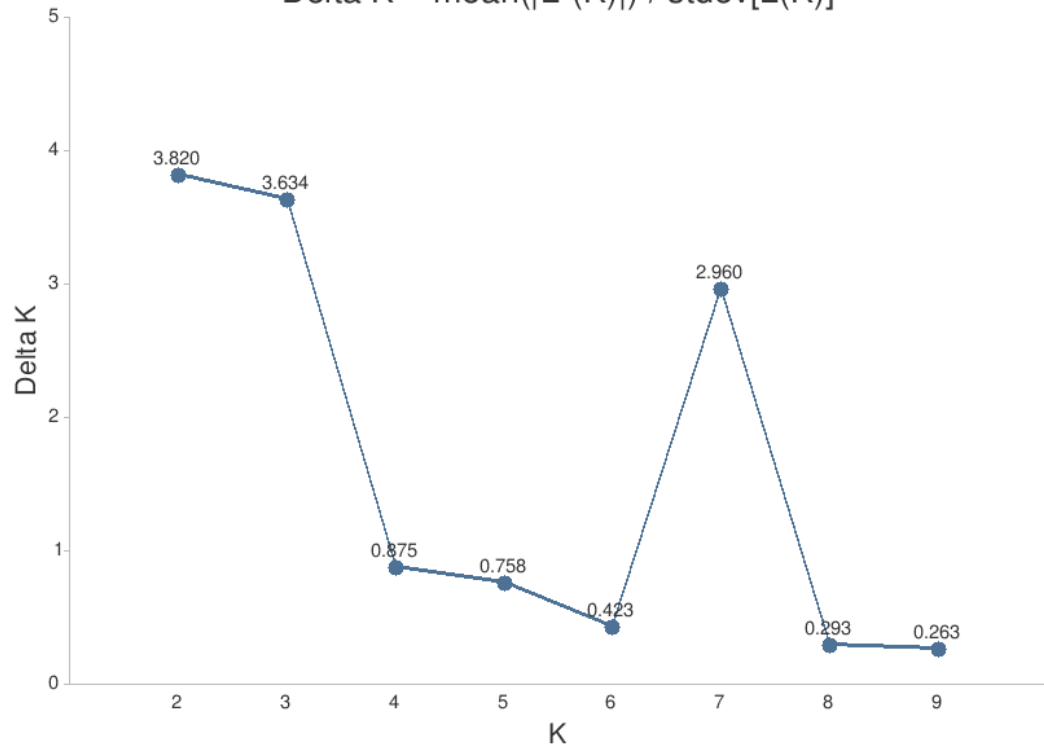

B)

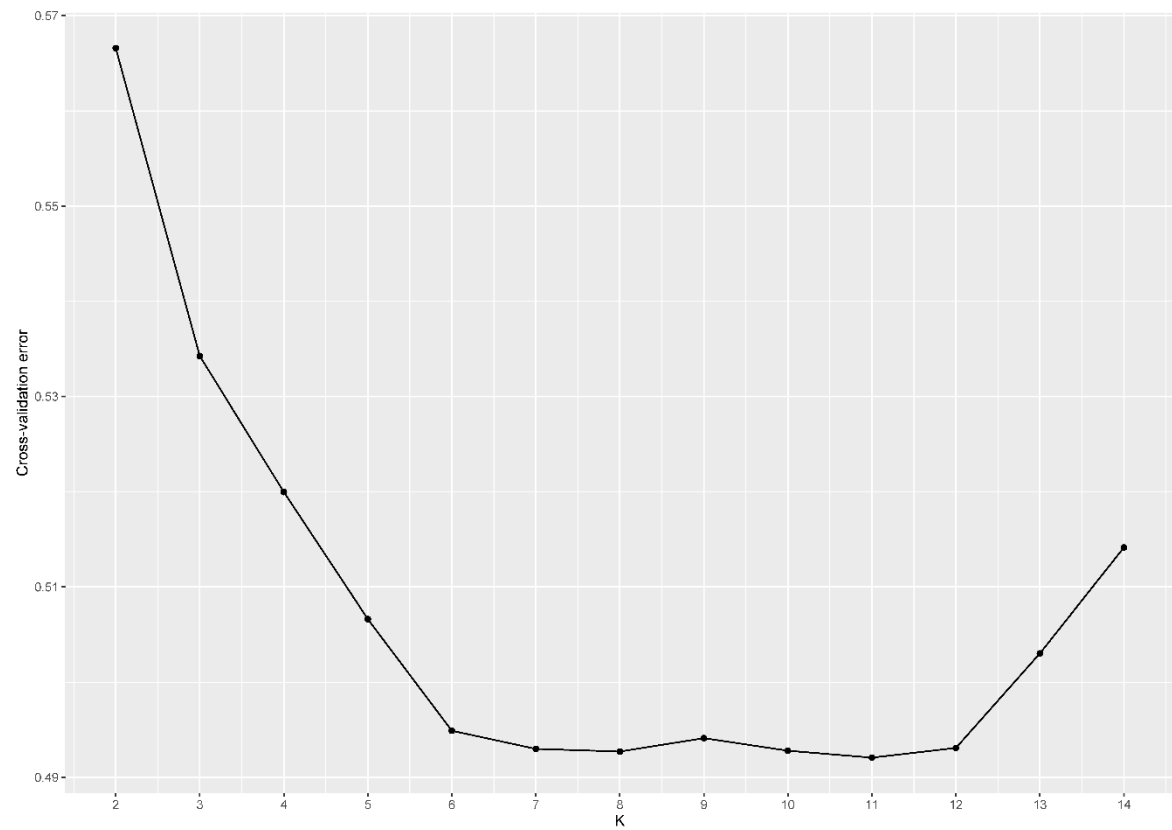

Supplement: Supplementary file 6 — Additional file 6: Figure S1. Optimal number of genetic clusters. Evanno’s statistic ΔK, showing a peak at the optimal number of clusters (A) and cross-validation (CV) error estimates of ADMIXTURE, with varying levels of K (B). [file 12711_2022_718_MOESM6_ESM.pdf]

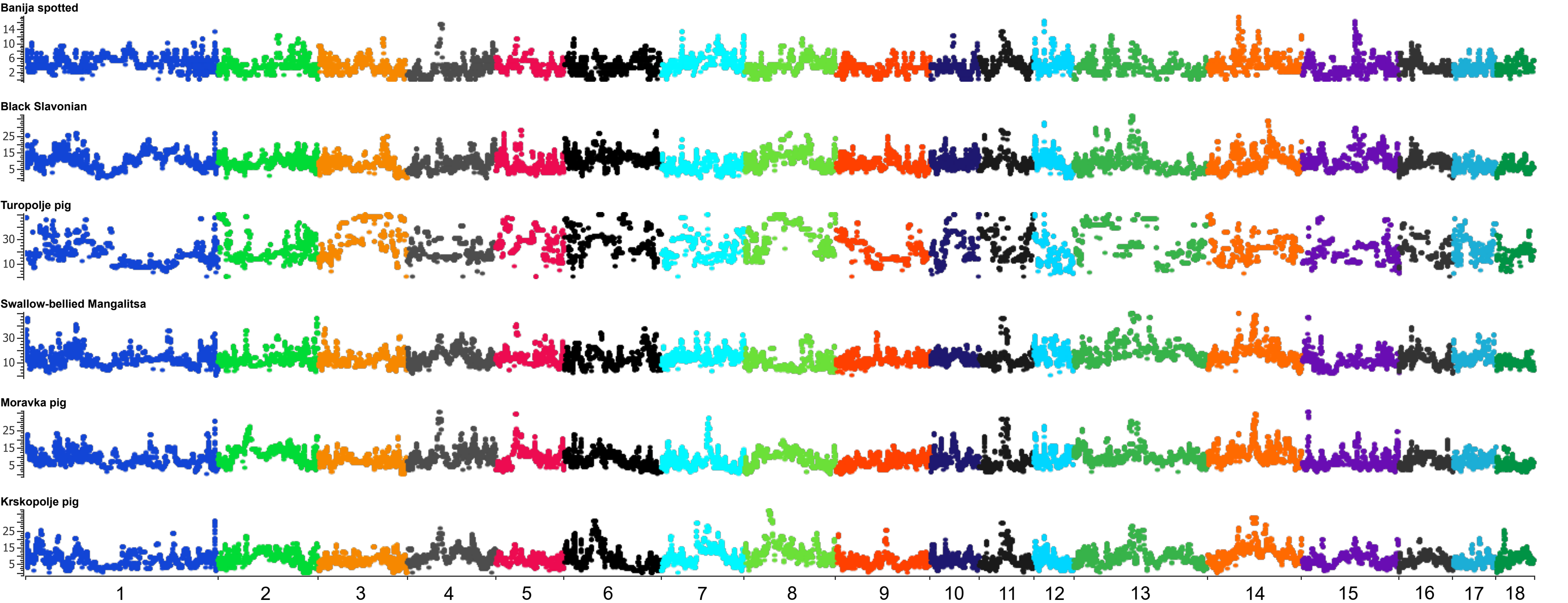

Supplement: Supplementary file 9 — Additional file 9: Figure S3. Manhattan plot of ROH islands. [file 12711_2022_718_MOESM9_ESM.pdf]
