# Supplementary material for: Genetic diversity and population structure of six autochthonous pig breeds from Croatia, Serbia, and Slovenia
Source: Genet Sel Evol. 2022 Apr 28;54:30. doi: 10.1186/s12711-022-00718-6 (PMC9052598; doi:10.1186/s12711-022-00718-6)
Supplement: Supplementary file 7 — Additional file 7: Table S6. Nei's genetic distances based on microsatellites and SNPs. [file 12711_2022_718_MOESM7_ESM.docx]

**Table S7**

Nei's genetic distances based on STR and SNP markers

|  | **Banija spotted** | **Black Slavonian** | **Krskopolje** | **Swallow-bellied Mangalitsa** | **Moravka** | **Turopolje** |
| --- | --- | --- | --- | --- | --- | --- |
| **STR** | | | | | | |
| **Banija Spotted** | 0.000 | - | - | - | - | - |
| **Black Slavonian** | 0.256 | 0.000 | - | - | - | - |
| **Krskopolje** | 0.257 | 0.307 | 0.000 | - | - | - |
| **Swallow-bellied Mangalitsa** | 0.474 | 0.394 | 0.464 | 0.000 | - | - |
| **Moravka** | 0.276 | 0.218 | 0.271 | 0.319 | 0.000 | - |
| **Turopolje** | 0.615 | 0.761 | 0.971 | 0.772 | 0.711 | 0.000 |
| **SNP** | | | | | | |
| **Banija spotted** | 0.000 | - | - | - | - | - |
| **Black Slavonian** | 0.068 | 0.000 | - | - | - | - |
| **Krskopolje** | 0.068 | 0.077 | 0.000 | - | - | - |
| **Swallow-bellied Mangalitsa** | 0.095 | 0.089 | 0.112 | 0.000 | - | - |
| **Moravka** | 0.061 | 0.066 | 0.067 | 0.083 | 0.000 | - |
| **Turopolje** | 0.144 | 0.149 | 0.163 | 0.148 | 0.145 | 0.000 |
