# Supplementary material for: Genetic diversity and population structure of six autochthonous pig breeds from Croatia, Serbia, and Slovenia
Source: Genet Sel Evol. 2022 Apr 28;54:30. doi: 10.1186/s12711-022-00718-6 (PMC9052598; doi:10.1186/s12711-022-00718-6)
Supplement: Supplementary file 8 — Additional file 8: Figure S2. Breed-pairwise shared identical-by-descent segments (> 7 Mb, 3-7 Mb, < 3Mbp). [file 12711_2022_718_MOESM8_ESM.pdf]

Banija spotted

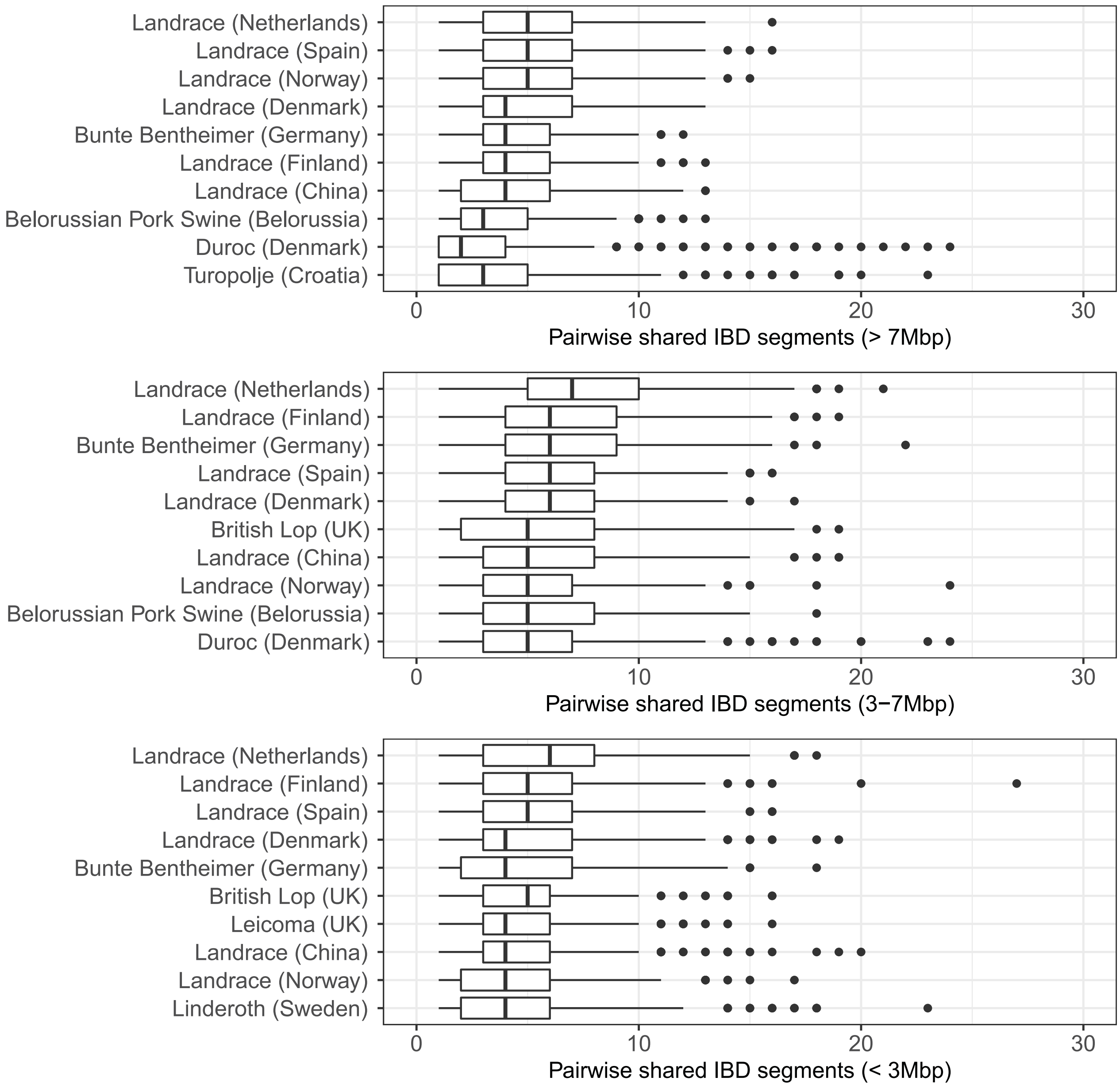

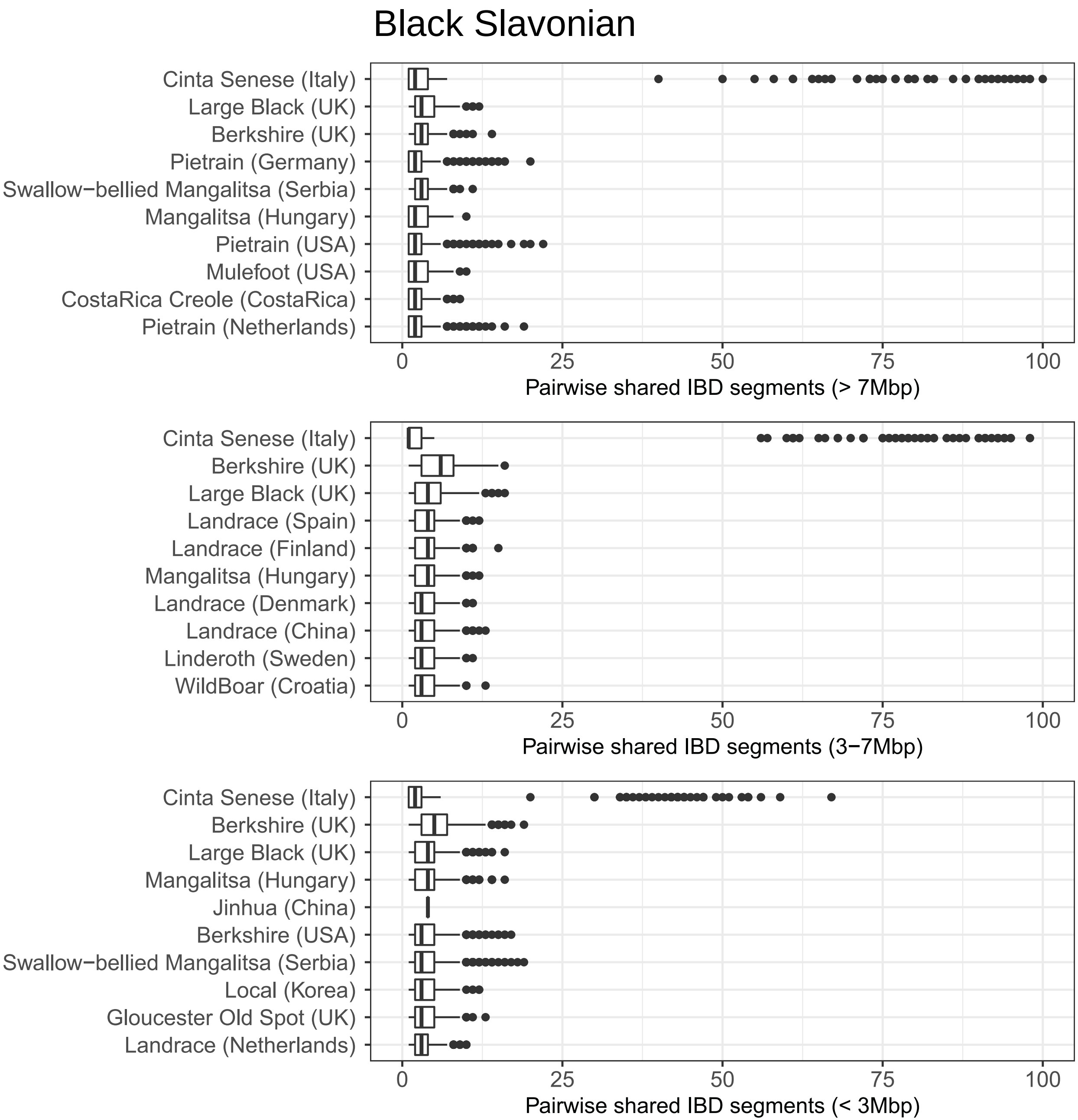
Black Slavonian
Cinta Senese (Italy)
Large Black (UK)
Berkshire (UK)
Pietrain (Germany)
Swallow−bellied Mangalitsa (Serbia)
Mangalitsa (Hungary)
Pietrain (USA)
Mulefoot (USA)
CostaRica Creole (CostaRica)
Pietrain (Netherlands)
0
25
50
75
100
Pairwise shared IBD segments (> 7Mbp)
Cinta Senese (Italy)
Berkshire (UK)
Large Black (UK)
Landrace (Spain)
Landrace (Finland)
Mangalitsa (Hungary)
Landrace (Denmark)
Landrace (China)
Linderoth (Sweden)
WildBoar (Croatia)
0
25
50
75
100
Pairwise shared IBD segments (3−7Mbp)
Cinta Senese (Italy)
Berkshire (UK)
Large Black (UK)
Mangalitsa (Hungary)
Jinhua (China)
Berkshire (USA)
Swallow−bellied Mangalitsa (Serbia)
Local (Korea)
Gloucester Old Spot (UK)
Landrace (Netherlands)
0
25
50
75
100
Pairwise shared IBD segments (< 3Mbp)

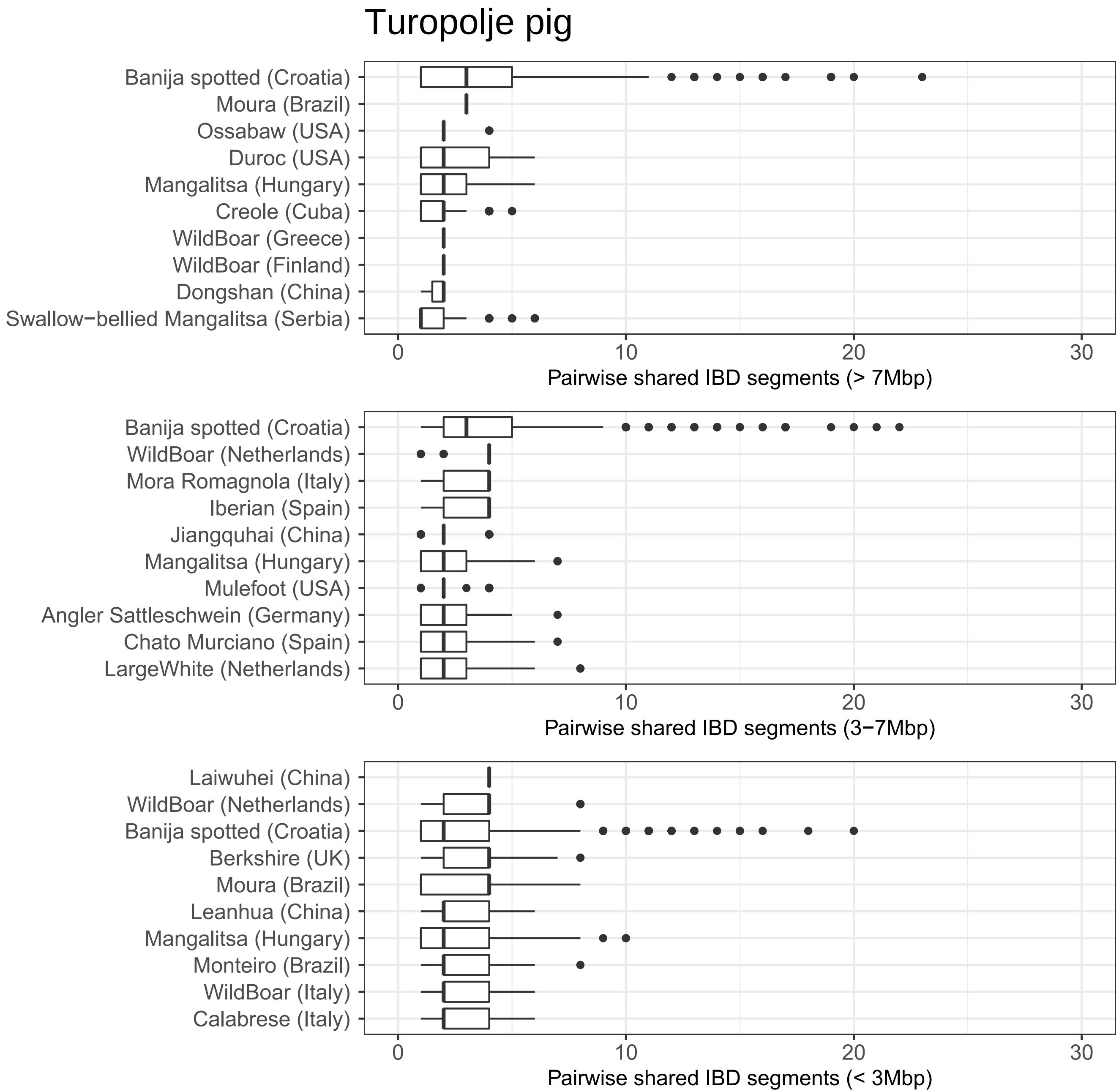
Turopolje pig
Banija spotted (Croatia)
Moura (Brazil)
Ossabaw (USA)
Duroc (USA)
Mangalitsa (Hungary)
Creole (Cuba)
WildBoar (Greece)
WildBoar (Finland)
Dongshan (China)
Swallow−bellied Mangalitsa (Serbia)
0
10
20
30
Pairwise shared IBD segments (> 7Mbp)
Banija spotted (Croatia)
WildBoar (Netherlands)
Mora Romagnola (Italy)
Iberian (Spain)
Jiangquhai (China)
Mangalitsa (Hungary)
Mulefoot (USA)
Angler Sattleschwein (Germany)
Chato Murciano (Spain)
LargeWhite (Netherlands)
0
10
20
30
Pairwise shared IBD segments (3−7Mbp)
Laiwuhei (China)
WildBoar (Netherlands)
Banija spotted (Croatia)
Berkshire (UK)
Moura (Brazil)
Leanhua (China)
Mangalitsa (Hungary)
Monteiro (Brazil)
WildBoar (Italy)
Calabrese (Italy)
0
10
20
30
Pairwise shared IBD segments (< 3Mbp)

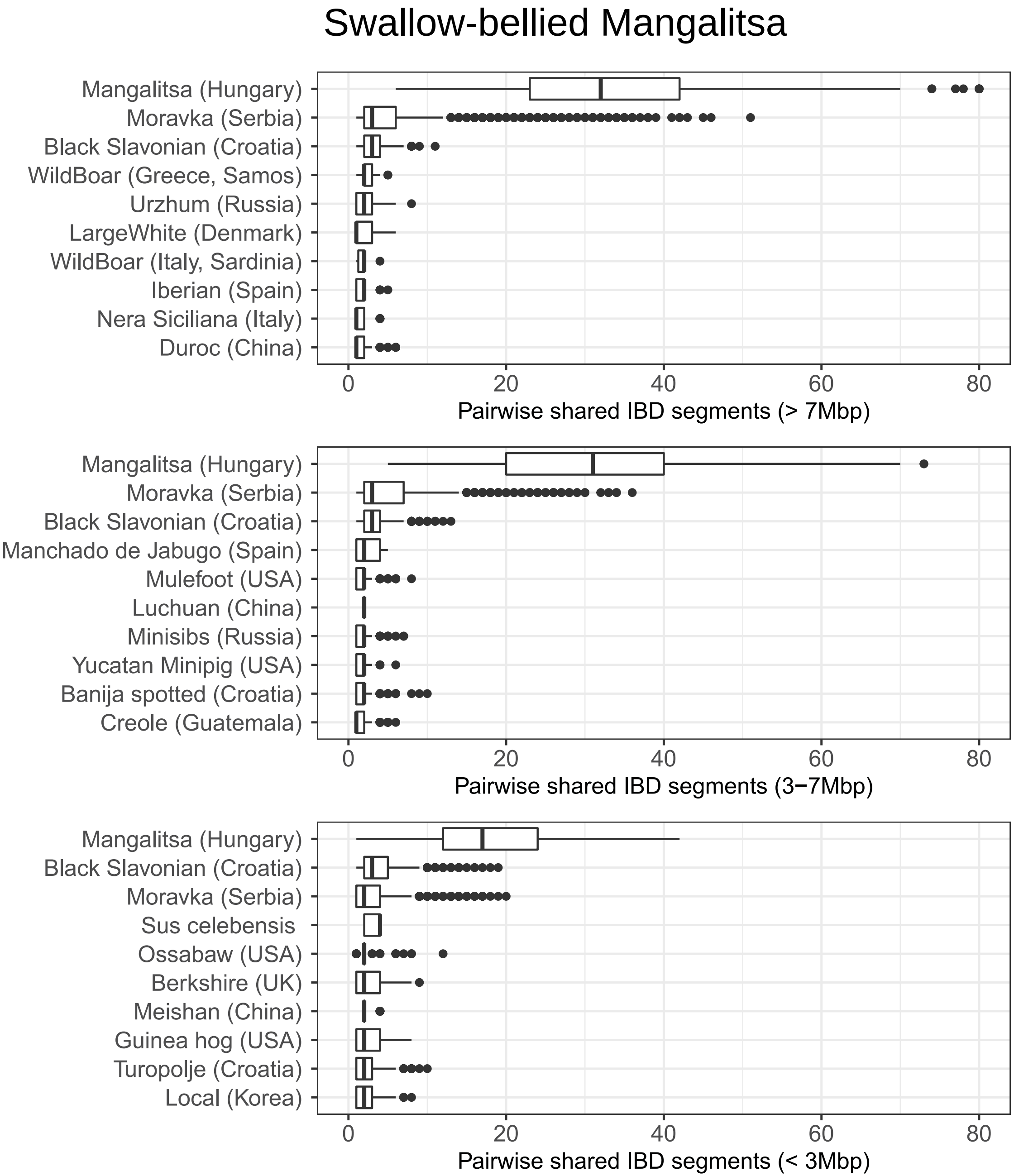
Swallow-bellied Mangalitsa
Mangalitsa (Hungary)
Moravka (Serbia)
Black Slavonian (Croatia)
WildBoar (Greece, Samos)
Urzhum (Russia)
LargeWhite (Denmark)
WildBoar (Italy, Sardinia)
Iberian (Spain)
Nera Siciliana (Italy)
Duroc (China)
0
20
40
60
80
Pairwise shared IBD segments (> 7Mbp)
Mangalitsa (Hungary)
Moravka (Serbia)
Black Slavonian (Croatia)
Manchado de Jabugo (Spain)
Mulefoot (USA)
Luchuan (China)
Minisibs (Russia)
Yucatan Minipig (USA)
Banija spotted (Croatia)
Creole (Guatemala)
0
20
40
60
80
Pairwise shared IBD segments (3−7Mbp)
Mangalitsa (Hungary)
Black Slavonian (Croatia)
Moravka (Serbia)
Sus celebensis
Ossabaw (USA)
Berkshire (UK)
Meishan (China)
Guinea hog (USA)
Turopolje (Croatia)
Local (Korea)
0
20
40
60
80
Pairwise shared IBD segments (< 3Mbp)

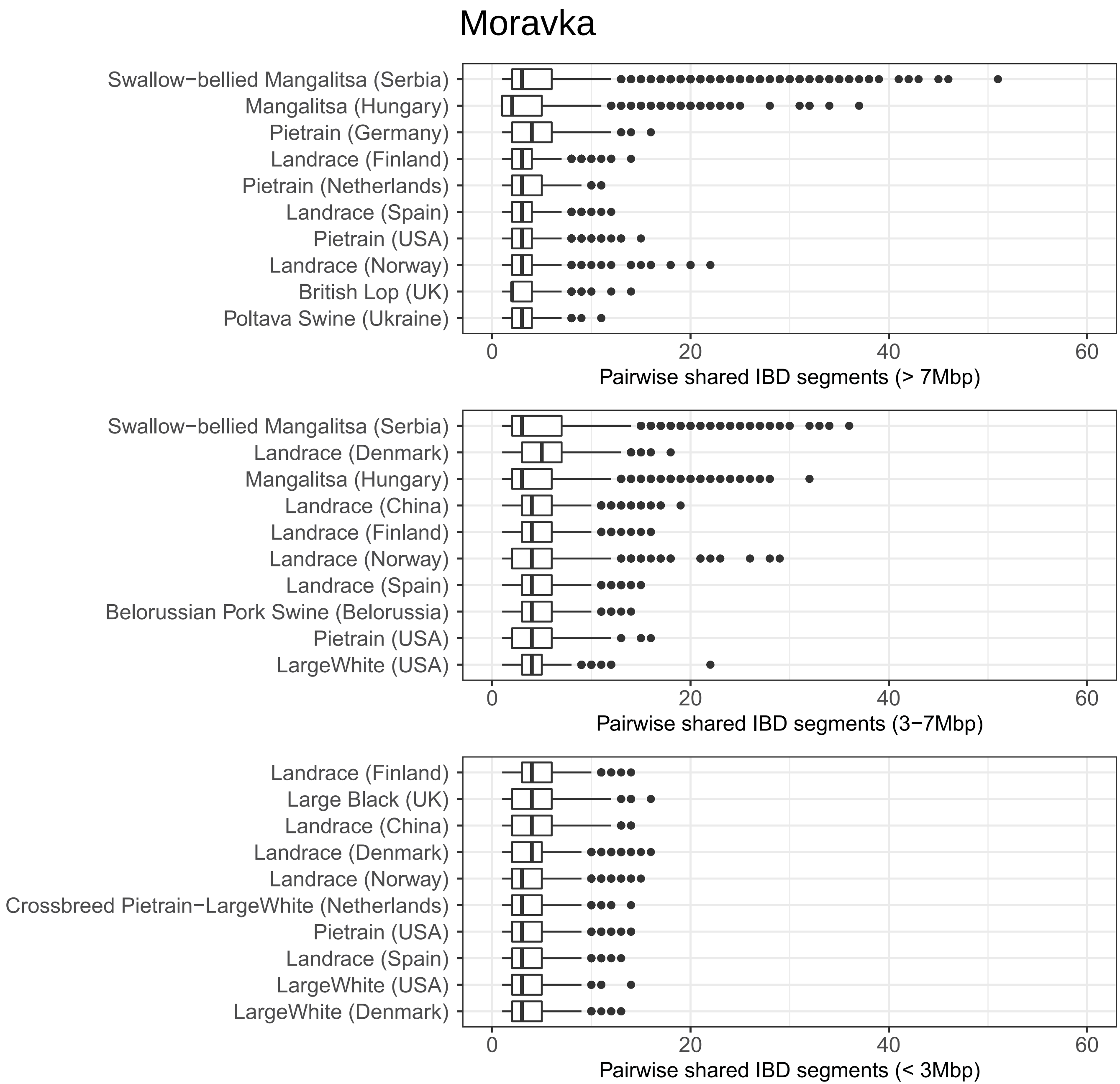

Moravka
Swallow−bellied Mangalitsa (Serbia)
Mangalitsa (Hungary)
Pietrain (Germany)
Landrace (Finland)
Pietrain (Netherlands)
Landrace (Spain)
Pietrain (USA)
Landrace (Norway)
British Lop (UK)
Poltava Swine (Ukraine)
0
20
40
60
Pairwise shared IBD segments (> 7Mbp)
Swallow−bellied Mangalitsa (Serbia)
Landrace (Denmark)
Mangalitsa (Hungary)
Landrace (China)
Landrace (Finland)
Landrace (Norway)
Landrace (Spain)
Belorussian Pork Swine (Belorussia)
Pietrain (USA)
LargeWhite (USA)
Pairwise shared IBD segments (3−7Mbp)
Landrace (Finland)
Large Black (UK)
Landrace (China)
Landrace (Denmark)
Landrace (Norway)
Crossbreed Pietrain−LargeWhite (Netherlands)
Pietrain (USA)
Landrace (Spain)
LargeWhite (USA)
LargeWhite (Denmark)
Pairwise shared IBD segments (< 3Mbp)

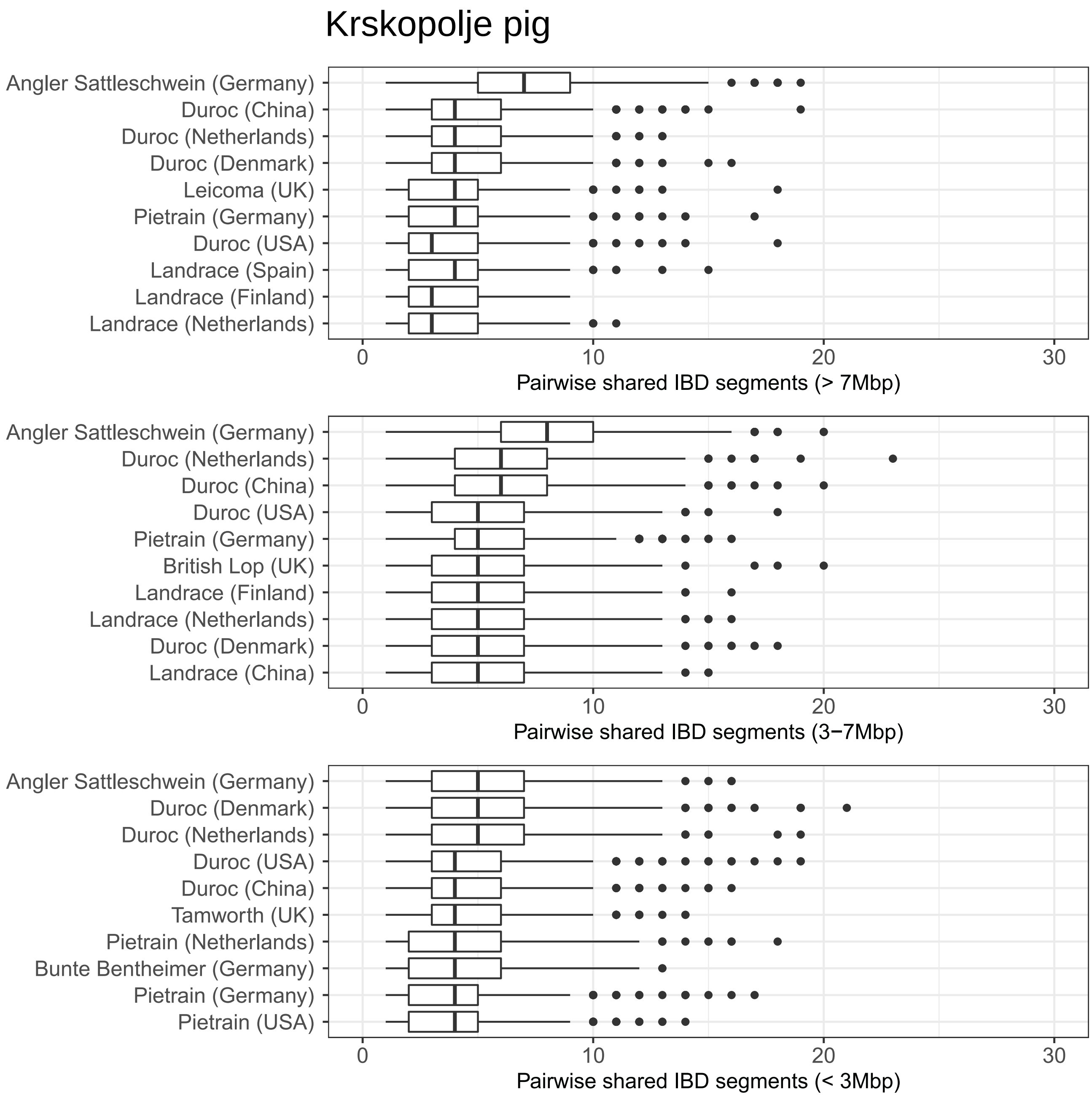

Krskopolje pig
Angler Sattleschwein (Germany)
Duroc (China)
Duroc (Netherlands)
Duroc (Denmark)
Leicoma (UK)
Pietrain (Germany)
Duroc (USA)
Landrace (Spain)
Landrace (Finland)
Landrace (Netherlands)
0
10
20
30
Pairwise shared IBD segments (> 7Mbp)
Angler Sattleschwein (Germany)
Duroc (Netherlands)
Duroc (China)
Duroc (USA)
Pietrain (Germany)
British Lop (UK)
Landrace (Finland)
Landrace (Netherlands)
Duroc (Denmark)
Landrace (China)
0
10
20
30
Pairwise shared IBD segments (3−7Mbp)
Angler Sattleschwein (Germany)
Duroc (Denmark)
Duroc (Netherlands)
Duroc (USA)
Duroc (China)
Tamworth (UK)
Pietrain (Netherlands)
Bunte Bentheimer (Germany)
Pietrain (Germany)
Pietrain (USA)
0
10
20
30
Pairwise shared IBD segments (< 3Mbp)
