# Supplementary material for: Genetic diversity and population structure of six autochthonous pig breeds from Croatia, Serbia, and Slovenia
Source: Genet Sel Evol. 2022 Apr 28;54:30. doi: 10.1186/s12711-022-00718-6 (PMC9052598; doi:10.1186/s12711-022-00718-6)
Supplement: Supplementary file 10 — Additional file 10: Table S7. ROH islands in the Banija spotted, Black Slavonian, Swallow-bellied Mangalitsa, Moravka and Krskopolje pig breeds [109–115, 117, 118, 120–136]. [file 12711_2022_718_MOESM10_ESM.docx]

**Table S10**

ROH islands in Banija spotted, Black Slavonian, Swallow-bellied Mangalitsa, Moravka and Krskopolje pig breeds

| **Chr** | **Start** | **End** | **Breed*** | **Gene(s)** | **Phenotype/function** | **References** |
| --- | --- | --- | --- | --- | --- | --- |
| 1 | 3128715 | 3477547 | Swallow-bellied Mangalitsa (90 %) | *PDE10A* | regulation of energy homeostasis, candidate target for obesity and diabetes | [120, 121] |
| 2 | 53594695 | 54076639 | Swallow-bellied Mangalitsa (90 %) | olfactory receptors (2L3, 2AJ1-like, 2L5-like, 2L2-like, 2L13-like, 2T12-like, 2M3-like) |  |  |
| 4 | 48746712 | 50945395 | Banija spotted (67 %) | *MMP16, CNBD1, CNGB3, CPNE3, RMDN1, WWP1, SLC7A13, ATP6V0D2* | biological regulation and metabolism, (ROH island in pig breeds from Poland) | [122] |
| 8 | 37395378 | 42765614 | Krskopolje (74 %) | *ATP10D, CORIN, NFXL1, CNGA1, NIPAL1, TXK, TEC, SLAIN2, SLC10A4, ZAR1, FRYL, OCIAD2, CWH43, DCUN1D4, LRRC66, SPATA18, USP46, RASL11B, SCFD2, LNX1, CHIC2, GSX2, PDGFRA, KIT, KDR, MAP9* | spermatogenesis (*SPATA18*), coat color (*KIT*) | [123, 118, 124, 117] |
| 11 | 33516188 | 36493548 | Swallow-bellied Mangalitsa (90 %) | *PCDH20* | establishment and maintenance of specific cell-cell connections in the brain; tameness in cats and silver foxes | [125-127] |
| 12 | 16536280 | 17032402 | Banija Spotted (71 %) | *ITGB3, MYL4, CDC27, KANSL1* | Selection signature and QTL (backfat and last rib) in Duroc pig population | [128] |
| 13 | 86046070 | 89510819 | Swallow-bellied Mangalitsa (98 %) | *PLOD2, PLSCR4, PLSCR1, PLSCR5, ZIC4, ZIC1, AGTR1, CPB1, GYG1, HLTF, HPS3, CP* | total number born, number born alive (*PLSCR4*), glycogen metabolism (*GYG1*), brown coat color (*HPS3*) | [109, 111] |
| 13 | 87535687 | 88187425 | Black Slavonian (73%) | *ZIC4, ZIC1, AGTR1, CPB1, GYG1, HLTF* | glycogen metabolism (*GYG1*) | [110] |
| 13 | 115266048 | 116503668 | Swallow-bellied Mangalitsa (92 %) | *TBL1XR1* | lipolysis in white adipose tissue | [129] |
| 14 | 47005710 | 47999414 | Swallow-bellied Mangalitsa (98 %) | *MTMR3, HORMAD2, LIF, OSM, CASTOR1, TBC1D10A, SF3A1, SEC14L2*,  *GAL3ST1, PES1, TCN2, SLC35E4, DUSP18, C5orf52, OSBP2, MORC2, SMTN, SELENOM, INPP5J, PLA2G3, LIMK2* | fatty acid metabolism (*PLA2G3*), regulation of the biosynthesis of cholesterol (*SEC14L2*), fat digestion and absorption (*PLA2G3*) | [112, 113] |
| 14 | 47243327 | 47881136 | Banija spotted (75%) | *OSM, CASTOR1, TBC1D10A, SF3A1, SEC14L2*,  *GAL3ST1, PES1, TCN2, SLC35E4, DUSP18, C5orf52, OSBP2, MORC2, SMTN* | regulation of the biosynthesis of cholesterol (*SEC14L2*), spermatogenesis and male fertility (*GAL3ST1*) (ROH island in Large White pig population) | [112, 114, 130] |
| 14 | 47895001 | 47999414 | Banija spotted (71%) | *SMTN, SELENOM, INPP5J, PLA2G3, LIMK2* | Fatty acid metabolism | [113] |
| 14 | 69814326 | 70113426 | Swallow-bellied Mangalitsa (94 %) | *CTNNA3* | intramuscular fat in longissimus dorsi muscle | [131] |
| 14 | 71463045 | 72418293 | Swallow-bellied Mangalitsa (96 %) | *PBLD, HNRNPH3, RUFY2, DNA2, SLC25A16, TET1, CCAR1, SNORD98, STOX1, SNORA70, DDX50, DDX21, KIFBP, SRGN, VPS26A, SUPV3L1, HKDC1, HK1* | carbohydrate metabolism (*HKDC1*) | [132, 133] |
| 14 | 90058648 | 91057395 | Black Slavonian (69 %) | *ERCC6, SLC18A3, CHAT, OGDHL, PARG, SNORA74, NCOA4, MSMB, ZFAND4, MARCHF8, ALOX5* | androstenone level (*NCOA4*), feet and leg soundness traits in pig (*ALOX5*) | [134, 135] |
| 15 | 8319700 | 8964248 | Moravka (70 %) | *ARHGAP15, KYNU* | tryptophan metabolism (*KYNU*) | [115] |
| 15 | 8319700 | 9255851 | Swallow-bellied Mangalitsa (92 %) | *ARHGAP15, KYNU* | tryptophan metabolism (*KYNU*) | [115] |
| 15 | 76813086 | 76879368 | Banija spotted (67 %) | *MYO3B, QRICH2* | Resistance to tuberculosis in dairy cattle (*MYO3B*) | [136] |

Genomic positions according to genome assembly Sscrofa11.1, * percentage of animals sharing ROH
