# Supplementary material for: Genetic diversity and population structure of six autochthonous pig breeds from Croatia, Serbia, and Slovenia
Source: Genet Sel Evol. 2022 Apr 28;54:30. doi: 10.1186/s12711-022-00718-6 (PMC9052598; doi:10.1186/s12711-022-00718-6)
Supplement: Supplementary file 11 — Additional file 11: Figure S4. Manhattan plot of signatures of selection (iHS). [file 12711_2022_718_MOESM11_ESM.pdf]

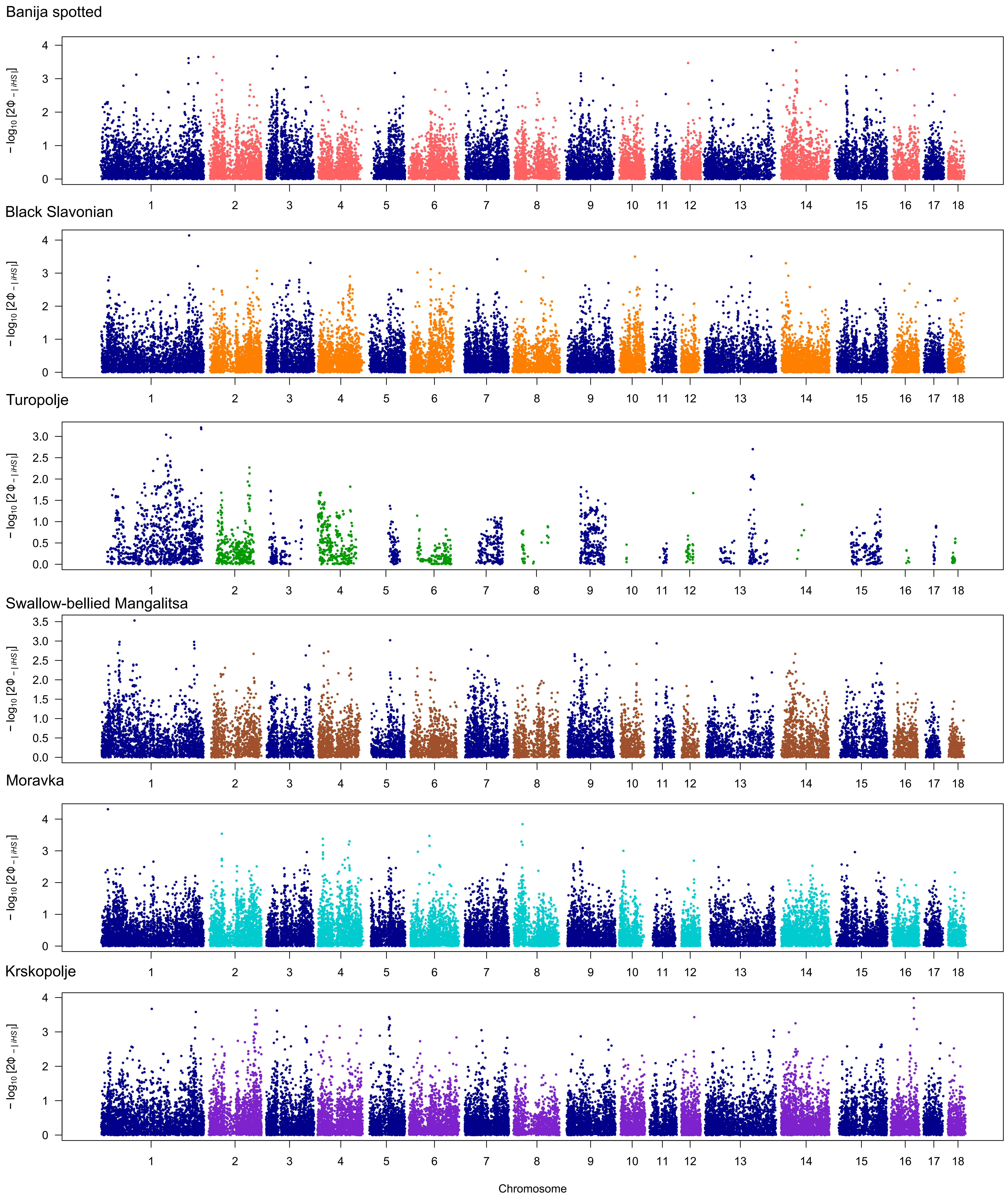

Banija spotted
Black Slavonian
Turopolje
Swallow-bellied Mangalitsa
Moravka
Krskopolje
$-\log_{10}[2\Phi_{-|iHS|}]$
1
2
3
4
5
6
7
8
9
10
11
12
13
14
15
16
17
18
Chromosome
