# Supplementary material for: Genetic diversity and population structure of six autochthonous pig breeds from Croatia, Serbia, and Slovenia
Source: Genet Sel Evol. 2022 Apr 28;54:30. doi: 10.1186/s12711-022-00718-6 (PMC9052598; doi:10.1186/s12711-022-00718-6)
Supplement: Supplementary file 12 — Additional file 12: Table S8. Signatures of selection in the Banija spotted, Black Slavonian, Turopolje pig, Swallow-bellied Mangalitsa, Moravka and Krskopolje pig breeds [60–82, 137–140]. [file 12711_2022_718_MOESM12_ESM.docx]

**Table S12**

Selection signatures

| **Chr.** | **Position** | **iHS** | **Gene** | **Phenotype** | **References** |
| --- | --- | --- | --- | --- | --- |
| **Banija spotted** | | | | | |
| 2 | 24847442 | 4.53 | *COMMD9*  *(upstream)* | cholesterol metabolism, cis-vaccenic acid (C18:1cis-11) in Alentejano pig, reproductive traits in cattle | [69, 70] |
|  |  |  | *LDLRAD3 (downstream)* | differentially expressed in Casertana and Large White transcripts in *longissimus*  muscle | [71] |
| 13 | 17108202 | 3.99 | *GADL1 (downstream)* | beef quality of *longissimus lumborum* muscle | [79] |
| 13 | 181025511 | 3.13 | *USP25 (upstream)* | tenderness | [72] |
| 14 | 43909637 | 3.54 | *SEZ6L* | disease resistance (porcine reproductive and respiratory syndrome, foot-and-mouth disease virus in cattle) | [137, 67] |
| 15 | 30546503 | 3.20 | *GLI2* | candidate gene for intramuscular fat content in pork (Duroc) | [73] |
| 15 | 29844258 | 3.56 | *CLASP1* | Warner-Bratzler shear force in Nelore beef cattle | [80] |
| 1 | 104241546 | 3.05 | *RAB27B (upstream)* | childhood body mass index (human) | [82] |
| **Black Slavonian** | | | | | |
| 3 | 68896427 | 3.12 | *BOLA3* | tick resistance in cattle | [68] |
| 3 | 94461983 | 3.09 | *PRKCE* | overall conformation (Duroc) | [74] |
| 6 | 32570824 | 3.41 | *TOX3* | loin depth | [75] |
| 10 | 42511563 | 3.32 | *ARHGAP12* | female fertility in cattle (QTL) | [60] |
| **Turopolje pig** | | | | | |
| 1 | 205738686 | 2.75 | *CNTLN* | Six-white-point coat color variance in the Diannan small-ear pigs | [65] |
| 1 | 123904362 | 2.75 | SLC24A5 | pigmentation | [66] |
| 5 | 65030604 | 2.89 | *NTF3 (downstream)* | cooking loss (beef) | [81] |
| **Swallow-bellied Mangalitsa** | | | | | |
| 1 | 95806521 | 3.57 | *ATP5F1A (upstream)* | post-thaw sperm quality in boars | [61] |
| 4 | 32527999 | 3.36 | *LRP12* | gluteus medius saturated fatty acid content | [76] |
| **Moravka** | | | | | |
| 8 | 27515272 | 3.93 | *ARAP2* | hematopoiesis | [78] |
| 8 | 30492334 | 3.55 | *WDR19* | semen quality and bull fertility | [62] |
| 9 | 42281094 | 3.45 | *CADM1* | meat quality traits in beef | [81] |
| **Krskopolje** | | | | | |
| 2 | 135413822 | 4.10 | *HSPA4* | spermatogenesis, sperm motility | [63, 64] |
| 4 | 20698525 | 3.63 | *EXT1* | levels of plasma triglycerides, C20:4(n-6)/C20:3(n-6) ratio | [77, 138] |
| 5 | 62481418 | 3.65 | *A2M* | involvement in early pregnancy establishment in pig, immune cell migration in pregnancy | [139, 140] |
